# LBX2 promotes colorectal cancer progression via the glycosylation and lactylation positive feedback

**DOI:** 10.1038/s41420-025-02888-w

**Published:** 2025-12-12

**Authors:** Yiwen Jiang, Lude Wang, Lin Chen, Kai Shen, Jie Chang, Shicong Zheng, Zewei Chen, Chenyang Ge, Min Yu, Shian Yu, Haiping Lin

**Affiliations:** 1 Department of General Surgery, Jinhua Central Hospital, Teaching Hospital of Mathematical Medicine College, Zhejiang Normal University, Zhejiang, China; 2 Department of Blood Transfusion, Affiliated Jinhua Hospital, Zhejiang University School of Medicine, Zhejiang, China; 3 Key Laboratory of Nutrition and Metabolism Research for Oncology, Affiliated Jinhua Hospital, Zhejiang University School of Medicine, Zhejiang, China; 4 Central Laboratory and Precision Medicine Center, Affiliated Jinhua Hospital, Zhejiang University School of Medicine, Zhejiang, China

**Keywords:** Cancer metabolism, Colorectal cancer

## Abstract

Colorectal cancer (CRC) ranks as a leading cause of cancer-related mortality worldwide, yet its molecular mechanisms remain incompletely understood. The transcription factor LBX2 regulates morphogenesis of multiple organ systems in vertebrates, yet its role in CRC progression remains poorly understood. In the study, we found that LBX2 knockdown suppresses CRC proliferation in vitro and in vivo. ChIP-seq/RNA-seq identifies GFPT2 as a direct transcriptional target of LBX2. The LBX2/GFPT2 axis elevates UDP-GlcNAc levels and O-GlcNAcylation, promoting Raptor T700 glycosylation. This modification enhances mTORC1 activation by strengthening Raptor-Rag interactions, accelerating glycolysis and lactate production. Accumulated lactate induces histone H4K12 lactylation, which further upregulates LBX2 transcription, forming a positive feedback loop. Clinically, high LBX2 expression correlates with elevated PET-CT SUVmax values (indicating hyperglycolysis) in CRC patients. Patient-derived organoids with high LBX2 show increased sensitivity to the GLUT1 inhibitor. LBX2 thus serves as both a metabolic driver and a potential biomarker for CRC-targeted therapies.

## Introduction

Colorectal cancer (CRC) is a common malignant tumor of the digestive tract. Globally, it ranks third in incidence among all malignant tumors and second in mortality, posing a significant burden on public health [1, 2]. Although the understanding of CRC initiation and progression has advanced significantly, the underlying molecular mechanisms remain highly complex and largely enigmatic [3, 4]. There is an urgent need to further investigate the molecular drivers of CRC progression in order to identify novel therapeutic targets and improve treatment strategies.

One such molecular driver is the transcription factor gene Ladybird homeobox 2 (LBX2), located on chromosome 2p13.1. LBX2 plays distinct yet critical roles in multiple developmental contexts. It is involved in the development of the male reproductive system (testis and epididymis) and is implicated in ovarian maturation and folliculogenesis, as indicated by its dynamic expression during follicular development [5, 6]. In zebrafish, it modulates both canonical and non-canonical Wnt signaling to influence myofilament gene expression and myogenesis, and further regulates cardiac development by affecting neural crest cell migration and cardiac septation [7–10]. Beyond its physiological roles, LBX2 has been implicated in tumorigenesis. Studies have shown that elevated LBX2 expression contributes to tumor progression and correlates with poor prognosis in various malignancies, including lung adenocarcinoma, ovarian cancer, and CRC [11–13]. However, the molecular mechanisms behind LBX2’s role in CRC remain poorly understood, and the regulatory pathways driving its upregulation in tumors are still unknown.

In addition to genetic factors, post-translational modifications such as glycosylation are crucial in the regulation of cancer. Glycosylation is an enzyme-mediated process that governs the biosynthesis, modification, and turnover of carbohydrate structures (glycans) [14, 15]. These glycans can exist either as free molecules or form covalent conjugates with proteins, lipids, and RNAs. One of the most prevalent and dynamic forms of glycosylation is O-GlcNAcylation, which involves the addition of UDP-N-acetylglucosamine (UDP-GlcNAc) to proteins [16]. This modification is critical for regulating cellular processes and is primarily driven by the hexosamine biosynthesis pathway (HBP), a key glucose metabolic branch [17, 18]. The HBP integrates multiple metabolic inputs to generate UDP-GlcNAc, which serves as the substrate for protein O-GlcNAcylation, thus linking cellular nutrient status to post-translational regulation.

Extensive studies have established that malignant transformation is associated with aberrant glycosylation pathways, which directly contribute to fundamental hallmarks of cancer, including tumor initiation and progression [19, 20]. O-GlcNAcylation can promote tumor progression by activating proliferation-related signaling pathways and regulating cell cycle progression[21, 22]. It can directly modulate cancer stem cell-like properties or indirectly regulate the expression and activity of other factors, thereby inducing cancer stem cell-like characteristics [23, 24]. In addition, O-GlcNAcylation has been reported to mediate cellular metabolic dysregulation in tumors. In breast cancer, O-GlcNAc Transferase (OGT) and O-GlcNAcylation modifications can inhibit tumor cell glycolysis [25]. In liver cancer, O-GlcNAcylation significantly enhances the stability of hypoxia-inducible factor 1α (HIF-1α), promoting a metabolic shift toward the Warburg effect [26]. However, in CRC, the regulatory role of O-GlcNAcylation in tumor metabolism remains understudied.

In this study, we confirmed that high LBX2 expression promotes proliferation and progression in CRC. Through ChIP-seq and RNA-seq, we identified that LBX2 regulates the transcriptional expression of Glutamine:Fructose-6-Phosphate Amidotransferase 2 (GFPT2), a key molecule in the HBP, which facilitates the synthesis of UDP-GlcNAc as a substrate for O-GlcNAcylation, thereby elevating overall O-GlcNAcylation levels in CRC cells. Specifically, O-GlcNAcylation of the regulatory-associated protein of mTOR (Raptor) component in mammalian target of rapamycin complex 1 (mTORC1) mediates mTORC1 activation and enhances downstream glycolytic metabolism, increasing intracellular lactate levels. This, in turn, regulates LBX2 gene transcription through lactylation of histone H4K12 (H4K12la) in the LBX2 chromatin region, forming a positive feedback loop that drives tumor progression.

## Results

### LBX2 promotes colorectal cancer proliferation in vitro and in vivo

**Fig. 1:**
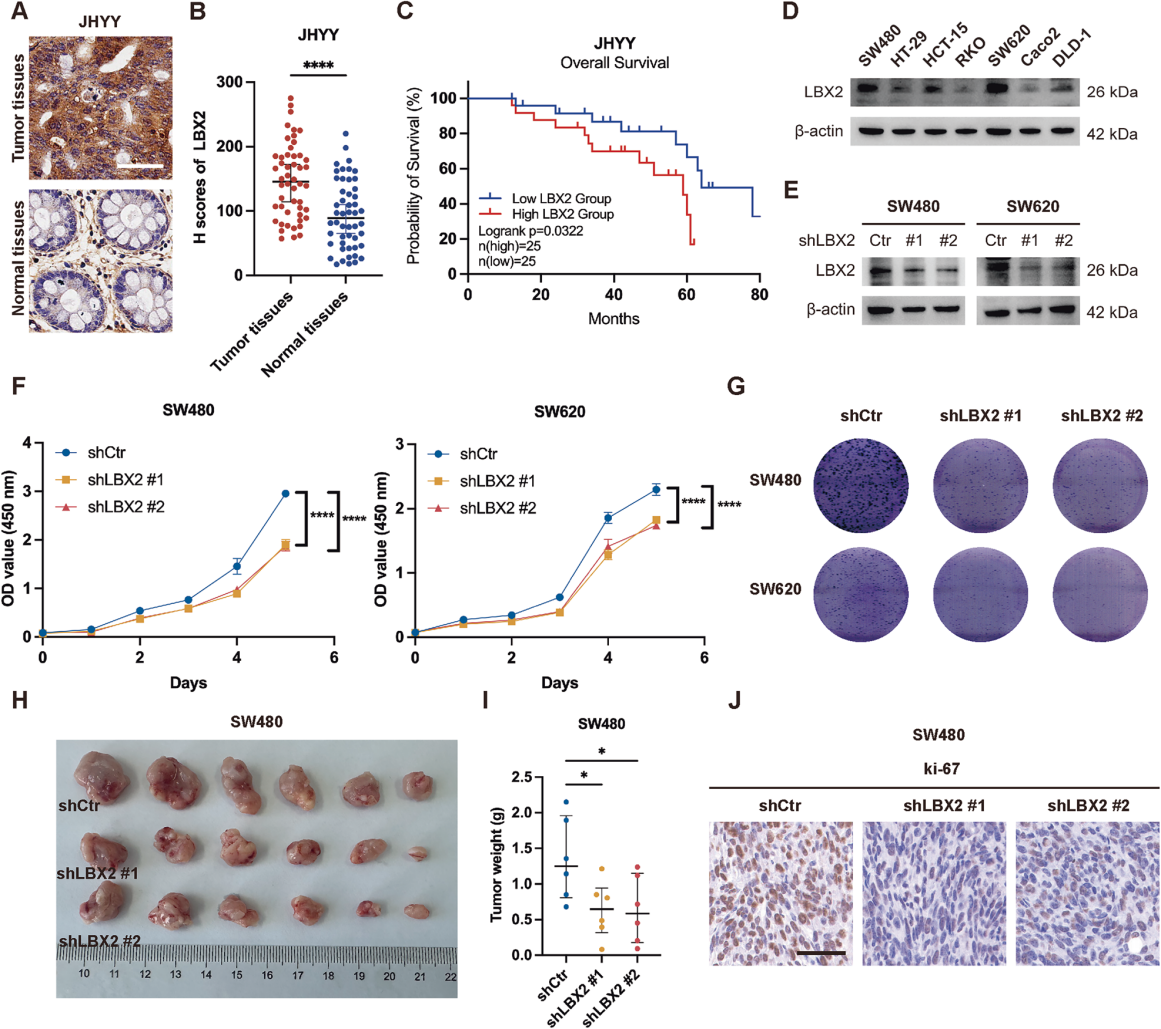
LBX2 is upregulated in colorectal cancer tissues and promotes tumor proliferation. Immunohistochemical (IHC) analysis (**A**) and quantification (**B**) of LBX2 expression in 50 paired tumor tissues and adjacent non-tumor tissues in CRC patients from the JHYY cohort (bar = 50 µm); **C** CRC patients from the JHYY cohort were stratified into LBX2 high-expression and low-expression groups based on the median IHC H-score in tumor tissues. Kaplan-Meier survival analysis and log-rank test were performed to compare overall survival (OS) outcomes between the two groups; **D** LBX2 expression levels in different CRC cell lines; **E** Western blot analysis to assess LBX2 knockdown efficiency in SW480 and SW620 cells with stable LBX2 knockdown; **F** CCK-8 assay to evaluate the proliferative capacity of SW480 and SW620 cells following LBX2 knockdown; **G** Colony formation assay to assess the proliferative capacity and clonogenic potential of SW480 and SW620 cells following LBX2 knockdown; **H** Assessment of SW480 proliferative capacity following LBX2 knockdown in a nude mouse subcutaneous tumor model (*n* = 6); **I** Following sacrifice, tumor weights were measured to assess changes in tumor mass after LBX2 knockdown; **J** Immunohistochemical analysis of Ki-67 expression levels in mouse tumor tissues between LBX2 knockdown and control groups (bar = 50 µm). *P < 0.05; ****P < 0.0001.

We first examined LBX2 expression differences between tumor and normal tissues in CRC, including colon adenocarcinoma (COAD) and rectal adenocarcinoma (READ), using datasets from TCGA and GEO (accessions GSE44076 and GSE87211). The results showed that LBX2 expression was significantly elevated in tumor tissues compared to normal tissues (Supplementary Fig. 1A, B). More importantly, high LBX2 expression was significantly associated with poor overall survival (OS) in CRC patients (Supplementary Fig. 1C, D). To validate these public database findings, we examined clinical samples from CRC patients at our center (JHYY cohort) (Supplementary Table 3). IHC analysis similarly demonstrated elevated LBX2 expression in tumor tissues compared to normal intestinal tissues (Fig. 1A, B). More importantly, in the JHYY cohort, high LBX2 expression in tumor tissues was similarly associated with poor prognosis (Fig. 1C).

To investigate the impact of LBX2 knockdown on CRC progression, we established stable LBX2-knockdown cell lines in SW480 and SW620 cells, which exhibit high LBX2 expression (Fig. 1D, E). Subsequent cell proliferation and colony formation assays revealed that LBX2 knockdown significantly inhibited the proliferative capacity of CRC cells (Fig. 1F, G, Supplementary Fig. 1E). In a subcutaneous tumor model of CRC in nude mice, we similarly observed that reduced LBX2 expression suppressed tumor growth, a finding corroborated by IHC analysis of Ki-67, a proliferation marker, in the subcutaneous tumors (Fig. 1H–J, Supplementary Fig. 1F). These results collectively demonstrate that LBX2 acts as an oncogene, promoting tumor progression in CRC.

### LBX2 transcriptionally activates GFPT2 expression

**Fig. 2:**
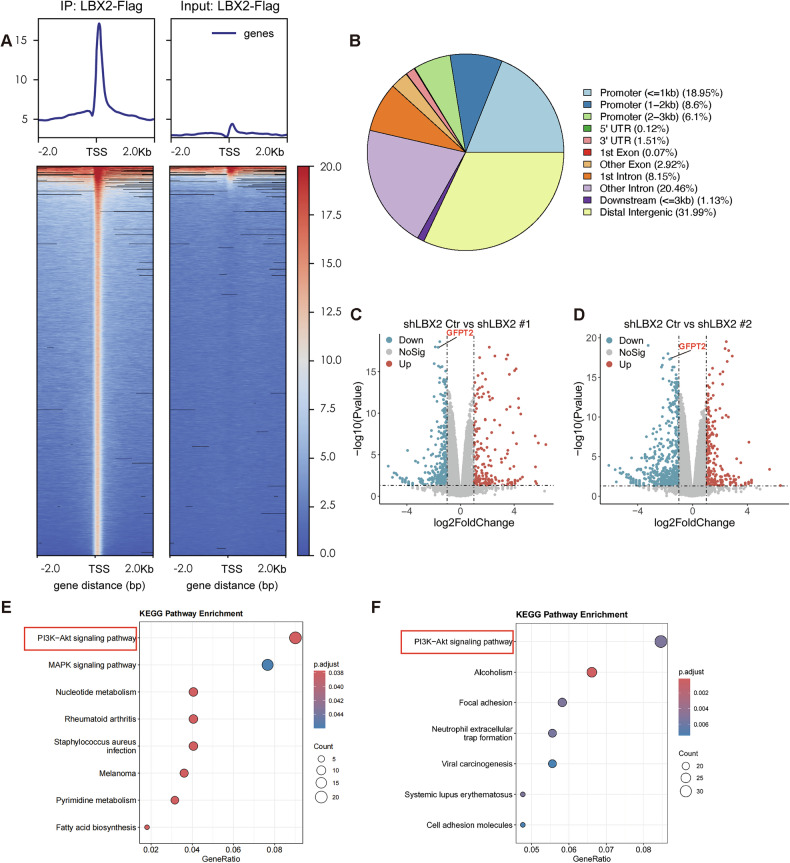
Integrative analysis of ChIP-seq and RNA-seq identifies LBX2 downstream targets and pathways. **A** ChIP-seq signal enrichment at transcription start sites (TSS) in SW480 cells transfected with LBX2-Flag and immunoprecipitated using a Flag antibody; **B** Distribution of ChIP-seq enriched fragments across different chromosomal regions; **C**, **D** Volcano plot showing the distribution of differentially expressed genes in RNA-seq analysis comparing SW480 shCtr vs. shLBX2 #1 and shCtr vs. shLBX2 #2; **E**, **F** KEGG pathway enrichment analysis of differentially expressed genes from RNA-seq comparing SW480 shCtr vs. shLBX2 #1 and shCtr vs. shLBX2 #2.

LBX2 mainly functions as a transcription factor within cells. To further explore its downstream target molecules and regulated pathways, we conducted ChIP-seq and RNA-seq analyses in SW480 cells. We initially performed ChIP-Seq to investigate DNA fragments interacting with the LBX2 protein, and results indicate that LBX2 primarily binds to distal intergenic and promoter regions in the genome of CRC cells (Fig. 2A, B, Supplementary Table 4). We subsequently performed RNA-seq to investigate the transcriptomic changes in SW480 cells following LBX2 knockdown (shLBX2 Ctr vs. shLBX2 #1 and shLBX2 Ctr vs. shLBX2 #2) (Fig. 2C, D, Supplementary Table 5). KEGG enrichment analysis of differentially expressed genes (DEGs) from shLBX2 Ctr vs. shLBX2 #1 and shCTR LBX2 Ctr vs. shLBX2 #2 revealed significant enrichment of the PI3K-Akt signaling pathway in both comparisons (Fig. 2E, F).

**Fig. 3:**
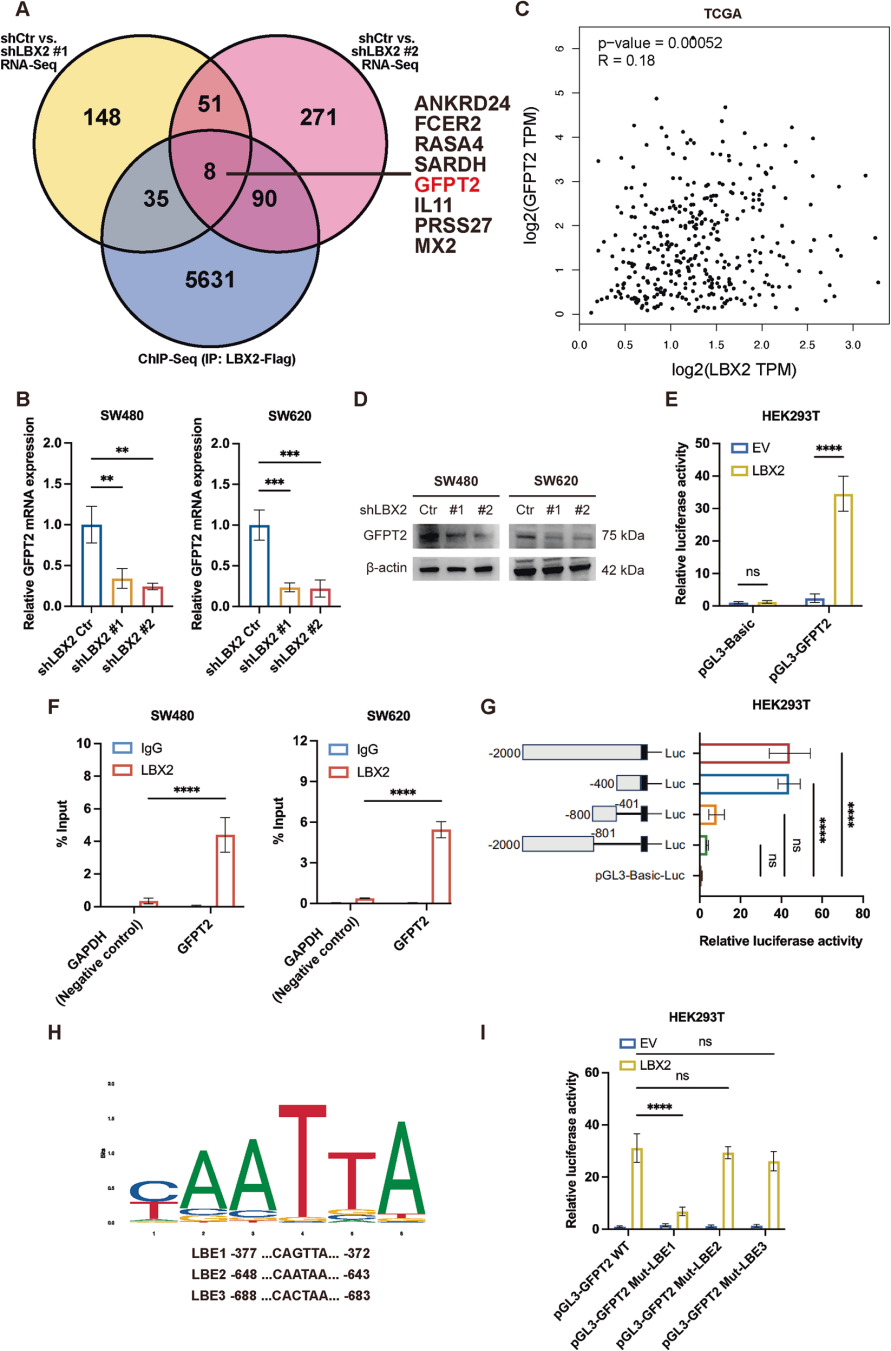
LBX2 transcriptionally activates GFPT2 expression. **A** Venn diagram showing the intersection of differentially expressed genes from RNA-seq (SW480 shCtr vs. shLBX2 #1 and shCtr vs. shLBX2 #2) and genes mapped from ChIP-seq enriched regions; **B** qRT-PCR analysis of GFPT2 mRNA expression levels in SW480 and SW620 cells transfected with shCtr, shLBX2 #1, and shLBX2 #2; **C** Correlation analysis of LBX2 and GFPT2 expression levels in the TCGA database**;** **D** Western blot analysis of GFPT2 protein expression levels in SW480 and SW620 cells transfected with shCtr, shLBX2 #1, and shLBX2 #2; **E** Dual-luciferase reporter assay to assess GFPT2 promoter activity (-2000 to +100 bp) following LBX2 overexpression in HEK293T cells; **F** ChIP-qPCR analysis of LBX2 enrichment at the GFPT2 promoter region in SW480 and SW620 cells; **G** Dual-luciferase reporter assay to assess the activity of different GFPT2 promoter deletion constructs following LBX2 overexpression in HEK293T cells; **H** JASPAR database identification of LBX2 binding motifs in the GFPT2 promoter region revealed three potential binding sites (LBE1: -377 to -372 bp; LBE2: −648 to −643 bp; LBE3: −688 to −683 bp); **I** Dual-luciferase reporter assay to assess the activity of different GFPT2 promoter mutants following LBX2 overexpression in HEK293T cells. ** *P* < 0.01; *** *P* < 0.001; **** *P* < 0.0001.

To identify potential downstream targets of the transcription factor LBX2, we integrated ChIP-seq and RNA-seq datasets (Fig. 3A), which revealed eight candidate genes. Among these, only GFPT2 and IL11 exhibited appreciable baseline expression in RNA-seq data, whereas the other six genes showed low expression levels (Supplementary Table 5). Subsequent qRT-PCR validation in CRC cells demonstrated that GFPT2 expression was significantly downregulated upon LBX2 knockdown, while IL11 levels remained largely unchanged (Fig. 3B, Supplementary Fig. 1G). Based on these findings, we selected GFPT2 for further investigation as a downstream effector of LBX2. By analyzing the TCGA database, we also observed a positive correlation between the RNA expression levels of LBX2 and GFPT2, suggesting that GFPT2 may serve as a downstream target of LBX2 transcriptional regulation (Fig. 3C).

To further validate the transcriptional regulation of GFPT2 by LBX2, we examined the changes in GFPT2 transcription and protein levels following LBX2 knockdown. The results demonstrated that LBX2 knockdown significantly reduced both the RNA and protein levels of GFPT2 in CRC cells (Fig. 3A, B). Subsequently, luciferase assay results confirmed that LBX2 overexpression significantly enhanced the transcriptional activity of the GFPT2 promoter region (Fig. 3C). ChIP-qPCR results further confirmed that LBX2 directly binds to the promoter region of GFPT2, thereby regulating its transcriptional activity (Fig. 3D).

To elucidate the specific region of the GFPT2 promoter regulated by LBX2, we constructed a series of truncated GFPT2 promoter plasmids using the pGL3-Luc vector. Luciferase assay results revealed that LBX2 modulates the transcriptional activity of the GFPT2 promoter within the -400 to +100 region (Fig. 3E). Using JASPAR to predict the specific binding sites of LBX2 on the GFPT2 promoter, we identified three potential LBX2 binding elements (LBEs) within the GFPT2 promoter region (Fig. 3F). By constructing corresponding mutants using site-directed mutagenesis, luciferase assay results further demonstrated that mutation of LBE1 significantly impaired LBX2’s ability to regulate the transcriptional activity of the GFPT2 promoter, whereas mutations in LBE2 and LBE3 had no notable effect (Fig. 3G).

### The LBX2/GFPT2 axis regulates intracellular O-GlcNAc levels through UDP-GlcNAc modulation

**Fig. 4:**
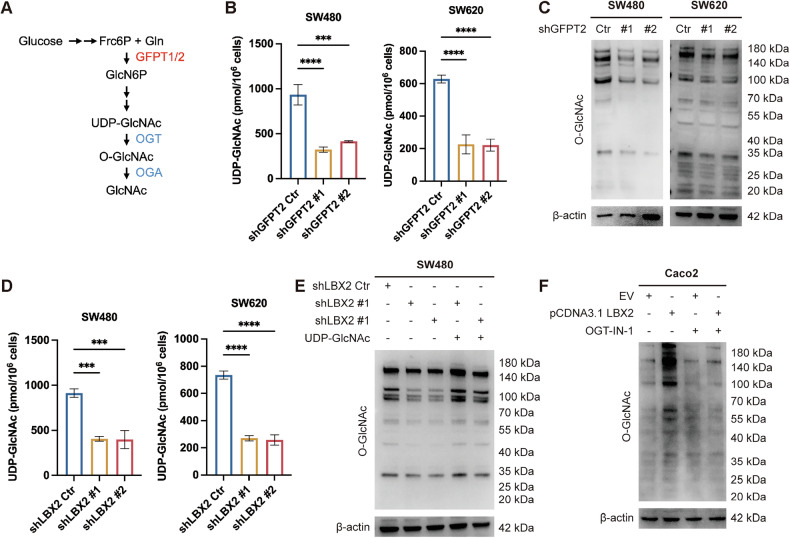
The LBX2/GFPT2 axis regulates intracellular O-GlcNAc levels through UDP-GlcNAc modulation. **A** Schematic diagram of the hexosamine biosynthesis pathway (HBP) pathway; **B** ELISA analysis of intracellular UDP-GlcNAc levels following GFPT2 knockdown in SW480 and SW620 cells; **C** Western blot analysis of total O-GlcNAc levels following GFPT2 knockdown in SW480 and SW620 cells; **D** ELISA analysis of intracellular UDP-GlcNAc levels following LBX2 knockdown in SW480 and SW620 cells; **E** Western blot analysis of total O-GlcNAc levels in SW480 cells following LBX2 knockdown and/or UDP-GlcNAc disodium supplementation (250 μM for 24 h); **F** Western blot analysis of total O-GlcNAc levels in Caco2 cells following LBX2 overexpression and/or OGT inhibition with OGT-IN-1 (2.0 μM for 24 h). ***P < 0.001; ****P < 0.0001.

GFPT2 catalyzes the conversion of glutamine and fructose-6-phosphate into glucosamine-6-phosphate, playing a pivotal role in the hexosamine biosynthetic pathway (HBP). This pathway generates UDP-N-acetylglucosamine (UDP-GlcNAc), a critical precursor for glycosylation modifications, such as O-GlcNAcylation, which profoundly influences protein function and cellular metabolic regulation (Fig. 4A). Knockdown of GFPT2 in CRC cells significantly reduces intracellular UDP-GlcNAc levels and overall O-GlcNAcylation, highlighting its critical role in modulating cellular glycosylation (Fig. 4B, C). Similarly, knockdown of LBX2 significantly decreases intracellular UDP-GlcNAc levels and overall O-GlcNAcylation, underscoring its regulatory role in cellular glycosylation processes (Fig. 4D, E).

OGT catalyzes the O-GlcNAc modification process by transferring UDP-GlcNAc to serine or threonine residues of target proteins, thereby regulating their function and cellular signaling. In Caco2 cells with relatively low LBX2 expression (Fig. 1D), we transfected an LBX2 overexpression plasmid and concurrently treated the cells with the OGT-specific inhibitor OGT-IN-1. The results revealed that OGT-IN-1 significantly attenuated the elevation of intracellular O-GlcNAcylation levels induced by LBX2 overexpression (Fig. 4F). Additionally, in LBX2-knockdown cells, supplementation with UDP-GlcNAc significantly restored intracellular O-GlcNAcylation levels (Fig. 4E). These findings indicate that the LBX2/GFPT2 axis regulates intracellular O-GlcNAc levels by controlling the production of UDP-GlcNAc, a critical substrate for glycosylation reactions.

### O-GlcNAcylation of Raptor modulates mTORC1-mediated glycolytic enhancement

**Fig. 5:**
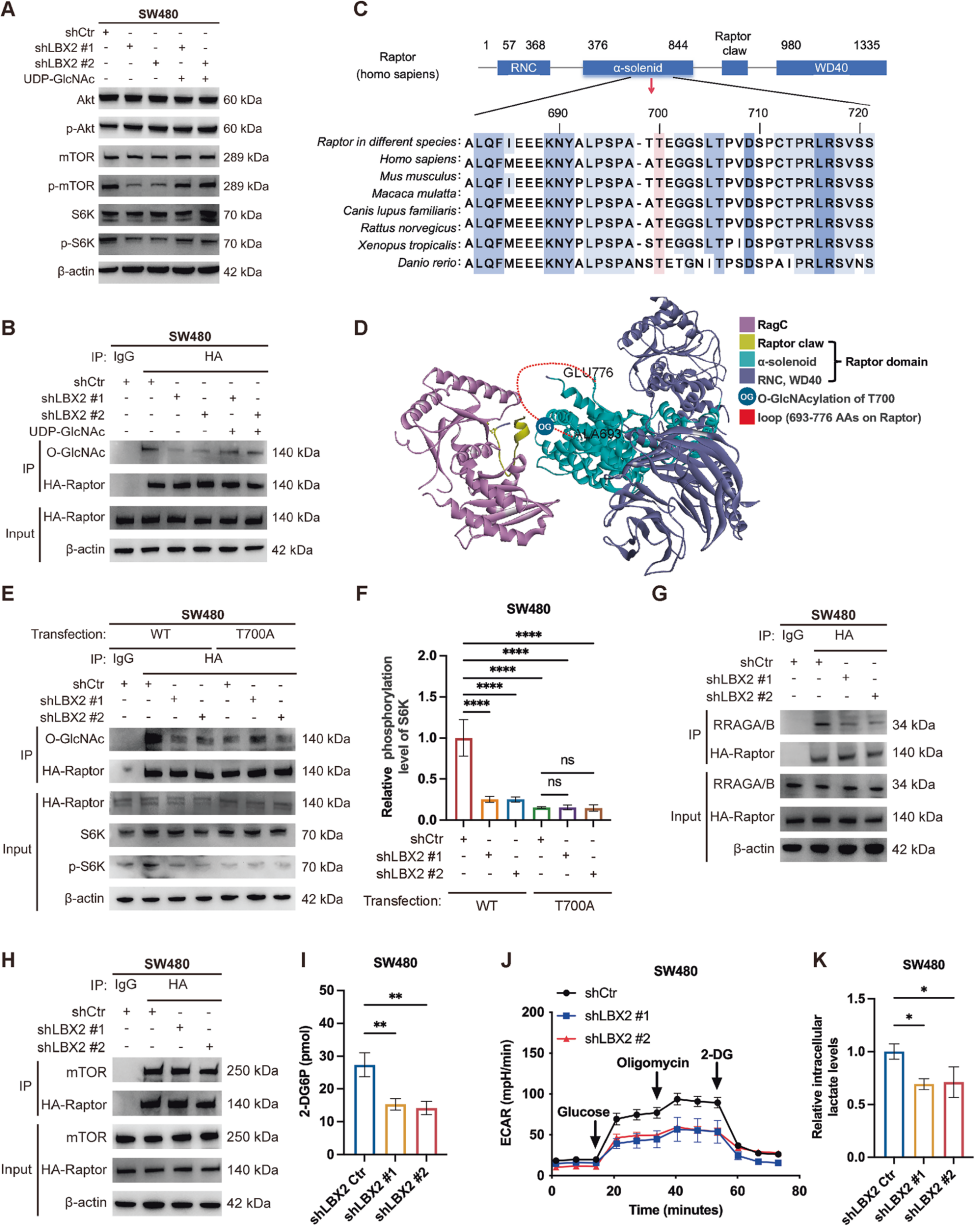
O-GlcNAcylation of Raptor modulates mTORC1-mediated glycolytic enhancement. **A** Western blot analysis of p-Akt/mTOR pathway activation following LBX2 knockdown and/or UDP-GlcNAc disodium supplementation (250 μM for 24 h) in SW480 cells; **B** Western blot analysis of SW480 cells with LBX2 knockdown and/or UDP-GlcNAc disodium supplementation (250 μM for 24 h), transfected with HA-Raptor plasmid, and subjected to immunoprecipitation using anti-HA antibody; **C** Conservation of residues 681–721 in Raptor. Pink: T700; **D** Structure of human Raptor-Rag (PDB: 6U62). Residue T700 is in a flexible region (693–776 aa) of Raptor; **E** Western blot analysis of SW480 cells with LBX2 knockdown, transfected with wild-type or T700D mutant HA-Raptor plasmid, and subjected to immunoprecipitation using anti-HA antibody; **F** The relative phosphorylation level of S6K in SW480 cells with LBX2 knockdown, transfected with wild-type or T700D mutant HA-Raptor plasmid, was calculated by quantifying the ratio of p-S6K to total S6K band intensity; **G**, **H** Western blot analysis of SW480 cells with LBX2 knockdown, transfected with HA-Raptor plasmid, and subjected to immunoprecipitation using anti-HA antibody; **I** ELISA analysis of intracellular 2-DG levels following LBX2 knockdown in SW480 cells; **J** Measurement of extracellular acidification rate (ECAR) in SW480 cells with LBX2 knockdown; **K** ELISA analysis of intracellular lactate levels following LBX2 knockdown in SW480 cells. *P < 0.05; **P < 0.01; ****P < 0.0001.

We further investigated how LBX2 regulates CRC proliferation through modulation of intracellular O-GlcNAcylation. Our RNA sequencing analysis of LBX2-knockdown cells revealed significant enrichment of the PI3K-Akt signaling pathway (Fig. 2E, F). Given that mammalian target of rapamycin (mTOR) is a key downstream effector of the PI3K-Akt pathway, we examined the effects of LBX2 knockdown on the PI3K-Akt-mTOR signaling cascade. The results demonstrated that LBX2 knockdown significantly suppressed the activation of mTOR and its downstream target ribosomal protein S6 kinase (S6K) (Fig. 5A). Surprisingly, upstream Akt signaling remained largely unaffected, suggesting that LBX2 likely regulates mTOR activation directly through modulation of mTORC1.

As a scaffold protein of mTORC1, Raptor directly interacts with Rags through its N-terminal HEAT repeat domain, thereby promoting the recruitment of mTORC1 to the lysosomal surface for activation. Previous studies have demonstrated that O-GlcNAcylation of Raptor regulates glucose-induced mTORC1 activation [27]. Accordingly, we hypothesize that in CRC, LBX2 similarly modulates mTORC1 activation by regulating O-GlcNAcylation of Raptor. Co-IP results confirmed that LBX2 knockdown reduces Raptor glycosylation levels, and this reduction can be restored by supplementation with UDP-GlcNAc (Fig. 5B). This change closely correlated with alterations in mTOR and S6K activity, supporting the conclusion that LBX2 controls Raptor O-GlcNAcylation by regulating UDP-GlcNAc availability.

We next sought to identify the specific O-GlcNAcylation site on Raptor that is regulated by LBX2. Protein-protein docking analysis revealed that residues 693-776 of Raptor form a flexible loop, which may mediate its interaction with Rags (Fig. 5C). Since O-GlcNAcylation primarily occurs on serine (S) or threonine (T) residues, we examined this loop for potential modification sites and found that T700 on Raptor is evolutionarily conserved (Fig. 5D). To verify that O-GlcNAcylation of Raptor at T700 mediates LBX2-induced mTORC1 activation in CRC, we transfected SW480 cells with either wild-type Raptor or a T700 mutant plasmid. We found that LBX2 knockdown reduced Raptor O-GlcNAcylation in cells expressing wild-type Raptor, but had no significant effect in those expressing the T700 mutant (Fig. 5E). Consistently, while LBX2 knockdown reduced intracellular mTOR activity in wild-type cells, this effect was not observed in T700 mutant-expressing cells (Fig. 5F).

To further validate whether LBX2-mediated O-GlcNAcylation affects the direct binding of Raptor to Rags, we conducted Co-IP experiments. The results revealed that LBX2 knockdown significantly inhibited the interaction between Raptor and Rags, while the Raptor-mTOR interaction remained unaffected (Fig. 5G, H). These findings suggest that LBX2 regulates mTORC1 activation by modulating the O-GlcNAcylation of Raptor at T700, thereby influencing the binding affinity between Raptor and Rags.

We next sought to determine how LBX2-regulated mTOR activity subsequently influences CRC proliferation. Given that mTORC1 serves as a central hub in regulating cellular glucose uptake, promoting both the expression and membrane localization of glucose transporters (GLUTs) and enhancing the glycolytic capacity of tumor cells, we further evaluated the impact of LBX2 on glucose uptake and glycolysis in CRC. Using the glucose analog 2-DG6P as a tracer for glucose uptake, we found that LBX2 knockdown significantly inhibited the ability of CRC cells to uptake extracellular glucose (Fig. 5I). Furthermore, by measuring the extracellular acidification rate, we observed that LBX2 knockdown also markedly suppressed the glycolytic capacity of CRC cells (Fig. 5J). The Warburg effect is a hallmark of cancer, and lactate is the primary product of anaerobic glycolysis. Therefore, we investigated the impact of LBX2 on lactate levels and found that LBX2 knockdown suppressed intracellular lactate levels in CRC cells (Fig. 5K). These results suggest that LBX2 regulates mTORC1 activity by modulating Raptor glycosylation, thereby promoting extracellular glucose uptake and intracellular glycolysis.

### Glycolytic enhancement-induced H4K12 lactylation epigenetically activates LBX2 transcriptional expression

The recently discovered histone lactylation represents a metabolite-sensing epigenetic mechanism. By conferring three-dimensional conformational changes to histone cores, this modification dynamically orchestrates transcriptional programs in response to cellular metabolic status. Given that the LBX2/GFPT2 axis regulates mTORC1 activation and glycolysis by modulating intracellular O-GlcNAc levels, we hypothesize that LBX2 may enhance the production of lactate, a glycolytic byproduct, thereby influencing intracellular histone lactylation levels.

**Fig. 6:**
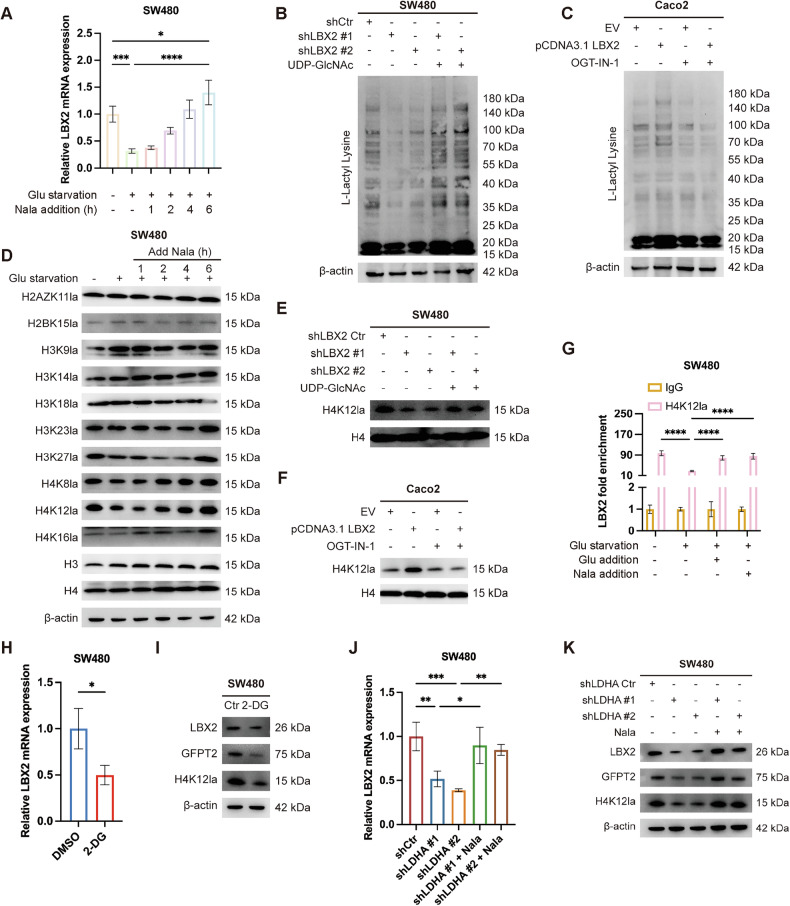
Glycolytic enhancement-induced H4K12 lactylation epigenetically activates LBX2 transcriptional expression. **A** qRT-PCR analysis of LBX2 mRNA expression levels in SW480 cells under glucose restriction (1.0 mM for 12 h) and exogenous sodium lactate supplementation (5.0 mM) for a specified duration; **B** Western blot analysis of total lactylation levels following LBX2 knockdown and UDP-GlcNAc disodium supplementation (250 μM for 24 h) in SW480 cells; **C** Western blot analysis of total lactylation levels following LBX2 overexpression and OGT-IN-1 treatment (2.0 μM for 24 h) in Caco2 cells; **D** Western blot analysis of site-specific histone lactylation in SW480 cells under glucose restriction and exogenous sodium lactate supplementation (5.0 mM) for a specified duration; **E** Western blot analysis of H4K12la following LBX2 knockdown and UDP-GlcNAc disodium supplementation (250 μM for 24 h) in SW480 cells; (**F**) Western blot analysis of H4K12la following LBX2 overexpression and OGT-IN-1 treatment (2.0 μM for 24 h) in Caco2 cells; **G** ChIP-qPCR analysis of H4K12la enrichment at the LBX2 promoter region in SW480 cells under glucose deprivation (1.0 mM for 12 h) and glucose (25.0 mM for 6 h) or sodium lactate (5.0 mM for 6 h) supplementation; **H** qRT-PCR analysis of LBX2 mRNA expression levels in SW480 cells treated with 2-DG (5.0 mM for 24 h); **I** Western blot analysis of LBX2 levels in SW480 cells treated with 2-DG (5.0 mM for 24 h); **J** qRT-PCR analysis of LBX2 mRNA expression levels following LDHA knockdown and sodium lactate supplementation (5.0 mM for 6 h) in SW480 cells; **K** Western blot analysis of LBX2 levels following LDHA knockdown and sodium lactate supplementation (5.0 mM for 6 h) in SW480 cells. *P < 0.05; **P < 0.01; ***P < 0.001; ****P < 0.0001.

To validate this hypothesis, we first examined LBX2 mRNA expression levels under glucose-deprived conditions. The results revealed that glucose deprivation significantly suppressed LBX2 mRNA expression, an effect that was reversed by supplementation with glucose (Supplementary Fig. 2A). Additionally, glucose significantly reduces intracellular lactate levels when depleted, while glucose supplementation restores these levels (Supplementary Fig. 2B). Correspondingly, lactate supplementation also reverses the decline in LBX2 mRNA expression induced by glucose deprivation (Fig. 6A).

We next investigated the impact of glucose deprivation on intracellular pan-lactylation levels. Consistent with changes in intracellular lactate levels, glucose deprivation reduced pan-lactylation levels, an effect that was similarly reversed by supplementation with glucose or sodium lactate (Supplementary Fig. 2C, D). We also investigated whether LBX2-mediated metabolic reprogramming affects pan-lactylation levels in CRC cells. Our results revealed that either knocking down LBX2 in SW480 cells with high LBX2 expression or overexpressing LBX2 in Caco2 cells with low LBX2 expression significantly altered cellular pan-lactylation levels (Fig. 6B, C). Furthermore, the reduction in pan-lactylation levels caused by LBX2 knockdown could be rescued by supplementation with the glycosylation substrate UDP-GlcNAc, while the increase in pan-lactylation levels induced by LBX2 overexpression was suppressed by the OGT inhibitor OGT-IN-1 (Fig. 6B, C).

Previous studies have shown that histone lactylation affects chromatin accessibility, thereby influencing transcription factor binding and the formation of the transcriptional initiation complex. Therefore, we further investigated the lactylation status of different lysine residues on various histones under glucose deprivation conditions. The results demonstrated that under glucose deprivation conditions, the lactylation levels of H3K23, H4K8, and H4K12 were significantly reduced (Fig. 6D, Supplementary Fig. 2E). Notably, only the lactylation of H4K12 was restored upon supplementation with glucose or sodium lactate (Fig. 6D, Supplementary Fig. 2E). Subsequent ChIP-qPCR results confirmed that H4K12 was specifically enriched in the LBX2 promoter region, with this enrichment significantly diminished under glucose deprivation and restored upon supplementation with glucose or sodium lactate (Fig. 6G). In contrast, H3K23 and H4K8 did not exhibit similar dynamic changes in the LBX2 promoter region (Supplementary Fig. 2F, G).

2-Deoxy-D-glucose (2-DG), a glucose analog, inhibits glycolysis by targeting hexokinase. Treatment of CRC cells with 2-DG significantly suppressed H4K12la and the expression of its target gene, LBX2 (Fig. 6H, I). Lactate dehydrogenase (LDH), particularly the LDHA homotetramer, is a critical enzyme in the glycolytic pathway, converting pyruvate to lactate. Transfection of CRC cells with LDHA shRNA markedly reduced H4K12la and LBX2 expression levels, and supplementation with sodium lactate restored the expression of these molecules (Fig. 6J, K, Supplementary Fig. 2H). These findings confirm that lactate-mediated H4K12 lactylation epigenetically regulates LBX2 transcriptional expression.

### Elevated LBX2 expression correlates with heightened glycolytic activity and increased sensitivity to glycolytic inhibitors

**Fig. 7:**
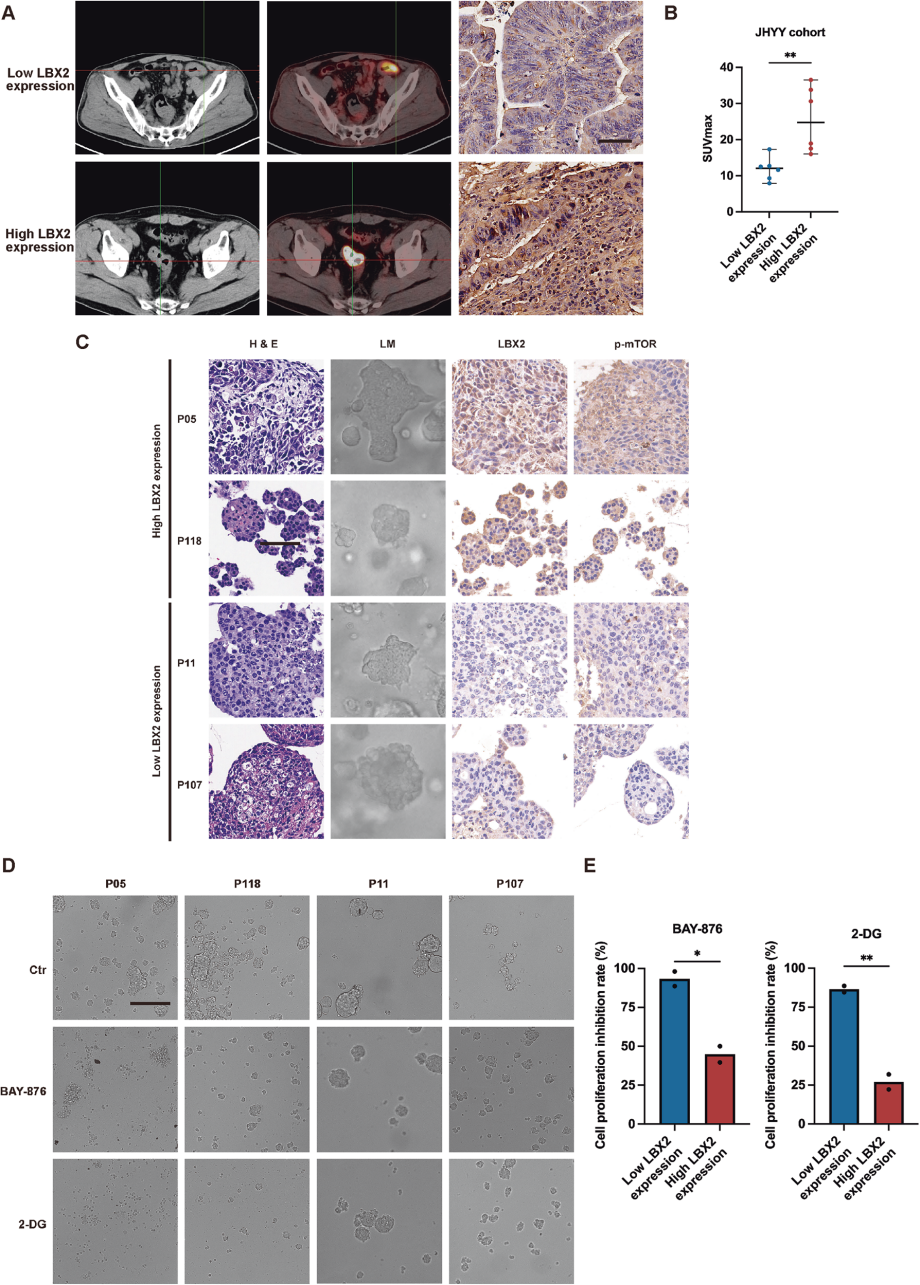
LBX2 expression levels correlate with glycolytic activity and sensitivity to glycolytic inhibitors. **A** PET and CT images of tumor sites on PET-CT scans from colorectal cancer patients with different LBX2 expression levels (bar = 50 µm); **B** SUVmax values of tumor sites on PET-CT scans from colorectal cancer patients with different LBX2 expression levels; **C** Light microscopy, H&E staining, and immunohistochemical images of LBX2 and p-mTOR in patient-derived colorectal cancer organoids with different LBX2 expression levels (bar = 25 µm); **D** Light microscopy showing the response of PDOs with different LBX2 expression levels to the Glucose Transporter 1 (GLUT1) inhibitor BAY-876 (20 μM for 48 h) or the glycolytic inhibitor 2-DG (20 mM for 48 h) (bar = 25 µm); **E** Viability of PDOs with different LBX2 expression levels treated with the BAY-876 (20 μM for 48 h) or the 2-DG (20 mM for 48 h). *P < 0.05; **P < 0.01.

To confirm that LBX2 regulates glycolysis in tumor tissues of CRC patients, we selected patients who had not received neoadjuvant therapy and had undergone PET-CT imaging prior to radical resection. We measured the SUVmax values of their tumor sites on PET-CT, a key semi-quantitative indicator that reflects the uptake of the radiotracer (¹⁸F-FDG) by the lesion, thereby indirectly assessing the level of tumor glucose metabolism. Based on LBX2 expression levels in tumor tissues, CRC patients were categorized into LBX2 high-expression and low-expression groups. The findings revealed that the SUVmax values, indicative of tumor glucose metabolism, were significantly higher in the LBX2 high-expression group compared to the low-expression group, suggesting that LBX2 high expression is associated with elevated glycolysis levels in CRC (Fig. 7A, B).

Organoids, as an advanced in vitro three-dimensional (3D) cell culture model, closely recapitulate the structure, function, genetic characteristics, and heterogeneity of tumors by mimicking the tumor microenvironment. They serve as an excellent model for studying tumor initiation, progression, and therapeutic responses. To validate the consistency between tumor tissues and organoid models, we established corresponding organoid models using tumor tissues from CRC patients. Correspondingly, elevated mTOR activation levels were observed in CRC organoids with high LBX2 expression (Fig. 7C).

The above findings demonstrate that high LBX2 expression promotes glycolysis by upregulating O-GlcNAcylation levels in tumors. Consequently, we hypothesize that targeting glucose metabolism may be more effective in CRC with high LBX2 expression. To validate this hypothesis, we treated CRC organoids with high or low LBX2 expression using either BAY-876 (a glucose transporter inhibitor) or 2-DG (a glycolytic inhibitor). Both BAY-876 and 2-DG showed significantly stronger proliferation inhibitory effects in CRC organoids with high LBX2 expression compared to those with low LBX2 expression (Fig. 7D, E). This finding suggests that LBX2 serves as an indicator of elevated glucose metabolism in CRC tumor tissues. More importantly, patients with high LBX2 expression may derive greater benefits from therapies targeting glucose metabolism.

## Discussion

**Fig. 8:**
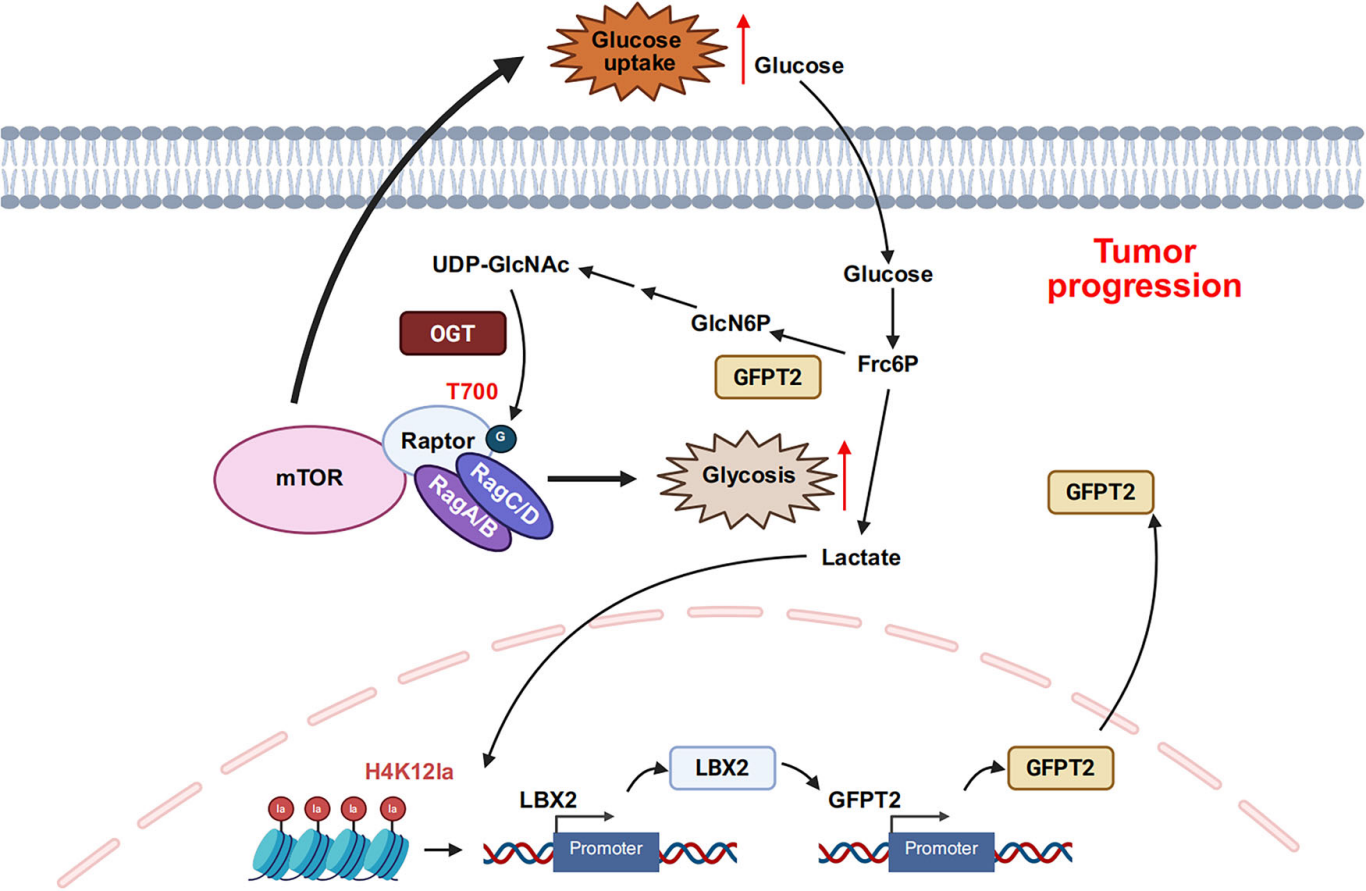
Schematic diagram of the study. LBX2 transcriptionally regulates GFPT2, modulating O-GlcNAcylation and Raptor T700 glycosylation in CRC cells, which alters mTORC1 activity, forming a regulatory axis with histone lactylation that promotes LBX2 upregulation.

This study reveals that in CRC, the transcription factor LBX2 orchestrates metabolic reprogramming by transcriptionally upregulating GFPT2—a key enzyme in the HBP that catalyzes the synthesis of GlcN-6-P, the precursor to UDP-GlcNAc [28]. This regulation elevates global O-GlcNAcylation levels, thereby enhancing glucose uptake and glycolytic flux in CRC. Mechanistically, O-GlcNAcylation at T700 of Raptor, a core component of mTORC1, facilitates its interaction with the Rag GTPase heterodimer on lysosomal membranes, leading to mTORC1 pathway activation and subsequent glycolytic promotion. Furthermore, lactate—a byproduct of enhanced glycolysis—accumulates intracellularly and induces H4K12la within the LBX2 chromatin region. This epigenetic modification upregulates LBX2 transcription, establishing a self-reinforcing LBX2-GFPT2-O-GlcNAcylation-mTORC1-lactate feedback loop that drives glycolytic metabolism and tumor progression (Fig. 8).

Our study is the first to demonstrate, through in vitro and in vivo experiments, that LBX2 promotes tumor proliferation and progression in CRC. In the LBX2-overexpressing CRC cell lines, stable LBX2 knockdown cell lines exhibited significantly reduced proliferation capacity. Similarly, in a nude mouse subcutaneous tumor model, we observed consistent effects of LBX2 on tumor progression. Consistent with our findings, studies in gastric cancer have indicated that LBX2-AS1 and LBX2 form a positive feedback loop, influencing the proliferation and apoptosis of gastric cancer cells [29]. However, the specific mechanisms by which LBX2 regulates gastric cancer cell proliferation remain to be fully elucidated Wang’s study.

To further elucidate the mechanisms by which LBX2 regulates CRC proliferation, we performed integrated ChIP-Seq and RNA-Seq analyses, given LBX2’s established role as a transcription factor [7]. The sequencing results revealed significant enrichment of the PI3K-Akt signaling pathway, and we identified GFPT2 as a potential downstream target transcriptionally regulated by LBX2. The regulation of GFPT2 by LBX2 was further confirmed through luciferase assays and ChIP-qPCR, which also identified the LBE1 site, located within the -400 to +100 promoter region of LBX2, as a critical regulatory element. GFPT2 is a key metabolic enzyme that plays a crucial role in the HBP, a glucose metabolism branch that generates UDP-GlcNAc—a critical substrate for protein O-GlcNAcylation, a post-translational modification involved in cellular signaling, stress response, and cancer progression. Our results confirmed that LBX2 knockdown reduces UDP-GlcNAc levels and O-GlcNAcylation in CRC cells. Conversely, the elevated O-GlcNAcylation induced by LBX2 overexpression can be reversed by an OGT inhibitor, where OGT is a key enzyme that catalyzes the addition of O-GlcNAc to serine and threonine residues of proteins [30].

To elucidate the role of LBX2-mediated O-GlcNAcylation in CRC proliferation, we analyzed sequencing data and observed significant enrichment of the PI3K-Akt signaling pathway. Intriguingly, while LBX2 knockdown markedly reduced phosphorylation of mTOR and its downstream target S6K, Akt phosphorylation was unaffected. Given prior reports that Raptor (a key subunit of mTORC1) is regulated by O-GlcNAcylation, we propose that LBX2 may modulate mTORC1 activity via GFPT2-dependent O-GlcNAcylation of Raptor [27]. As anticipated, LBX2 regulates the O-GlcNAcylation of Raptor at T700, thereby enhancing Raptor-Rag interactions and promoting mTORC1 activation. mTORC1, a central hub for cellular metabolic regulation, integrates nutrient, energy, and growth factor signals to balance anabolism and catabolism [31]. It activates HIF-1α, upregulating GLUT1/3 and glycolytic enzymes, thereby enhancing tumor cell glycolysis while suppressing mitochondrial oxidative phosphorylation, favoring glycolysis [32]. Our results also demonstrated that LBX2 regulates extracellular glucose uptake, glycolytic capacity, and lactate production in CRC cells.

To further investigate the causes of LBX2’s aberrant expression in CRC, we recognized that lactate, beyond being a terminal product of glycolysis, has recently been identified as a key metabolic signaling molecule [33, 34]. Studies have revealed that lactate can serve as a donor to modify lysine residues on histones through enzymatic or non-enzymatic mechanisms, resulting in histone lactylation, which directly influences gene expression [35]. For instance, global lactylome profiling in pancreatic cancer identified H3K18 lactylation (H3K18la) as a key epigenetic event that transcriptionally upregulates ACAT2. This cascade disrupts oxidative phosphorylation, creating a self-sustaining lactate loop that is ultimately implicated in cholesterol-mediated immunosuppression within the tumor microenvironment [36]. Similarly, in ovarian cancer, histone lactylation at H4K12 (H4K12la) activates the RAD23 Homolog A (RAD23A) enhancer to facilitate DNA damage repair, ultimately driving resistance to niraparib [37].

Given that LBX2 promotes lactate production by regulating glycolysis, we hypothesized that lactylation alters the chromatin structure of LBX2, thereby affecting its transcriptional activity. As expected, LBX2 mRNA levels were modulated by glucose and lactate concentrations, and changes in LBX2 gene expression influenced overall cellular lactylation levels. Through screening common histone lactylation sites, we identified H4K12la as a site affected by LBX2-mediated metabolic reprogramming of glycolysis. Consequently, our study uncovered a positive feedback axis in CRC: LBX2 transcriptionally upregulates GFPT2, a key metabolic enzyme in the HBP; GFPT2 modulates the glycosylation of Raptor in the mTORC1 complex, thereby regulating mTORC1 activation; mTORC1, as a central metabolic regulator, drives metabolic reprogramming to enhance glycolysis; and the resulting lactate accumulation influences LBX2 transcriptional activity through lactylation. This establishes a mechanism in CRC wherein glycosylation and lactylation reciprocally regulate each other.

Finally, we explored the clinical relevance of this positive feedback axis. PET-CT detects gamma rays emitted by a radioactive tracer to map metabolic activity, with ¹⁸F-FDG used to measure glucose metabolism, where higher ¹⁸F-FDG uptake correlates with elevated glycolytic activity [38]. Correspondingly, in CRC clinical samples, we found a positive correlation between LBX2 expression levels and the SUVmax of ¹⁸F-FDG on PET-CT. More importantly, we investigated whether LBX2 could serve as a biomarker for guiding the use of glycolysis-related inhibitors. Using patient-derived organoids, a 3D cell culture model that recapitulates in vivo tumor characteristics and cellular heterogeneity [39, 40], we validated organoids with high LBX2 expression exhibited greater sensitivity to GLUT1 inhibitors. Our study suggests that LBX2 serves as a marker of elevated glycolytic activity in CRC, indicating that patients with high LBX2 expression may benefit more from therapies targeting glucose metabolism.

## Methods

### Cell line culture

The CRC cells (SW480, HT-29, HCT-15, RKO, SW620, Caco2, and DLD-1) and HEK293T cells were purchased from the Chinese Academy of Sciences Shanghai Cell Bank and cultured in Dulbecco’s Modified Eagle Medium (DMEM) supplemented with 10% fetal bovine serum at 37 °C in a humidified atmosphere containing 5% CO₂. Each cell line was authenticated by Short Tandem Repeat (STR) profiling and confirmed to be free of mycoplasma contamination.

### Lentiviral packaging and stable cell line construction

For lentivirus packaging, HEK293T cells were seeded in 6-well plates at 60–70% confluency. The lentiviral transfer plasmid, along with the packaging plasmids psPAX2 and pMD2.G, was co-transfected using Lipofectamine 3000 at a ratio of 3:2:1 (μg plasmid DNA). The medium was replaced with fresh complete DMEM 6–8 h post-transfection. Viral supernatants were collected at 48 and 72 h, filtered through a 0.45-μm PVDF membrane, and either used immediately or stored at −80 °C. To establish stable cell lines, CRC cells were infected with lentivirus in the presence of 10 μg/mL polybrene and selected with appropriate antibiotics (e.g., puromycin, 1–5 μg/mL) for 3–5 days.

### Patient-Derived Organoids (PDOs) culture

Patient-derived organoids were established from resected tumor tissues of CRC patients with written informed consent, following institutional ethical guidelines. Tumor tissues were minced and digested in a solution containing 2 mg/mL collagenase IV, 0.2 mg/mL hyaluronidase, 0.2 mg/mL DNase I, and 100 µg/mL Primocin. The digested tissue was embedded in Matrigel and cultured in Advanced DMEM/F-12 medium supplemented with 1X GlutaMAX™, 1X HEPES, 10 mM nicotinamide, 1X B27 supplement, 1 mM N-acetylcysteine, 10 nM gastrin, 500 nM A83-01, 3 µM SB202190, 50 ng/mL recombinant human EGF, 100 ng/mL recombinant human noggin, 500 ng/mL recombinant human R-spondin-1, and 100 µg/mL Primocin. Organoids were maintained at passages below 30 and cultured at 37 °C in a humidified 5% CO₂ atmosphere.

### Cell viability assay

CRC cells were seeded in 96-well plates at a density of 1 × 10³ cells per well and allowed to adhere overnight. At each time point (Day 0–5), 10 μL of CCK-8 reagent was added to each well, followed by incubation for 2 h at 37 °C. The absorbance at 450 nm was measured using a microplate reader (BioTek, USA). Wells containing medium and CCK-8 reagent without cells served as blanks.

Cell viability of PDOs was assessed using the CellTiter-Blue® Cell Viability Assay (Promega, Madison, WI, USA) following the manufacturer’s protocol. Briefly, cells were seeded in 96-well plates at an appropriate density and allowed to adhere overnight. After treatment with the indicated conditions, 20 μL of CellTiter-Blue® reagent was added to each well containing 100 μL of culture medium, and the plates were incubated for 4 hours at 37 °C. A total of three independent biological replicates were performed. Fluorescence intensity was measured using a microplate reader (BioTek, USA) with an excitation wavelength of 560 nm and an emission wavelength of 590 nm. Wells containing medium and CellTiter-Blue® reagent without cells served as blanks.

### Colony formation assay

CRC cells were seeded into 6-well plates at a density of 1000 cells/well and cultured under standard conditions for 21 days. The medium was refreshed every 3–4 days to maintain nutrient supply. After 21 days, cells were gently washed twice with PBS, fixed with 4% paraformaldehyde for 15 min at room temperature, and stained with 0.5% crystal violet in methanol for 30 min. The plates were then rinsed with distilled water and air-dried. Colonies containing ≥50 cells were counted manually under a light microscope. A total of three independent biological replicates were performed. For statistical analysis, three independent experiments were performed with duplicate wells for each condition.

### Cell Protein Lysis and Immunoblotting Assay

For cell protein extraction, cells were lysed in RIPA buffer supplemented with a protease and phosphatase inhibitor cocktail. Immunoblotting followed standard protocols, with target protein signals detected using an enhanced chemiluminescence (ECL) system. β-Actin served as the loading control, and the antibodies used are detailed in the Supplementary Table 1.

### Nude mouse xenograft model

Four-week-old BALB/c nude mice were obtained from GemPharmatech (Nanjing, China) and randomly classified into three groups. Each mouse was subcutaneously injected on the right ventral flank with 3 × 10⁶ SW480 cells stably expressing shCtr, shLBX2 #1, or shLBX2 #2. Approximately 25 days post-inoculation, mice were euthanized, and tumors were excised, weighed, and photographed. The sample size (*n* = 6 in each group) used in the animal experiments is consistent with those commonly employed in comparable studies in this research field. All animal care and experimental procedures adhered to ethical guidelines and were approved by the Experimental Animal Welfare and Ethics Committee of Jinhua Hospital, Zhejiang University School of Medicine, Zhejiang, China (NO. AL-JHYY202508).

### Clinical sample collection

Fresh CRC tissues and paired adjacent normal tissues (≥5 cm from the tumor margin) were obtained from CRC patients who underwent surgical resection at Jinhua Hospital, Zhejiang University School of Medicine. This study was approved by the Ethics Committee of Jinhua Hospital, Zhejiang University School of Medicine. Written informed consent was obtained from all participants prior to sample collection (NO. 2022-Ethical review-203).

### Immunohistochemistry (IHC) and Quantification

Paraffin-embedded tissue sections (3 μm thick) were deparaffinized in xylene and rehydrated through a graded ethanol series. Antigen retrieval was performed by heating sections in citrate buffer (pH 6.0) at 95 °C for 20 minutes. Endogenous peroxidase activity was blocked with 3% hydrogen peroxide for 10 minutes. Sections were then incubated with primary antibodies (listed in the Supplementary Table 1) overnight at 4 °C, followed by incubation with horseradish peroxidase (HRP)-conjugated secondary antibodies for 1 hour at room temperature. Immunoreactive signals were visualized using 3,3’-diaminobenzidine (DAB) substrate, and sections were counterstained with hematoxylin. Slides were imaged using a light microscope. For quantification, the H-score was calculated based on the intensity and percentage of positively stained cells.

### Chromatin Immunoprecipitation (ChIP) Assay

ChIP-Seq was performed by CloudSeq Biotech (Shanghai, China) using the GenSeq® Chromatin Immunoprecipitation (ChIP) Kit (GenSeq Inc.) according to the manufacturer’s protocol. Chromatin was immunoprecipitated as described previously [41]. Briefly, cells were cross-linked with formaldehyde, lysed, and the chromatin was enzymatically and mechanically sheared into fragments corresponding to 1–5 nucleosomes by Micrococcal Nuclease digestion in conjunction with sonication. Subsequent immunoprecipitation was conducted using anti-Flag antibodies or control IgG. The yield of enriched DNA was quantified using the Quanti-IT™ fluorescence assay (Thermo Fisher Scientific). Sequencing libraries were prepared with the GenSeq® Rapid DNA Library Prep Kit (GenSeq Inc.) following the manufacturer’s instructions. Library quality was assessed using an Agilent 2100 Bioanalyzer (Agilent Technologies), followed by high-throughput 150 bp paired-end sequencing on an Illumina NovaSeq platform. Enriched DNA was further validated by qRT-PCR, with primer sequences listed in the Supplementary Table 2.

### RNA-Sequencing (RNA-Seq)

Total RNA was extracted from samples using TRIzol reagent (Thermo Fisher Scientific). RNA integrity was assessed using an Agilent 2200 TapeStation system, with only samples exhibiting an RNA Integrity Number (RIN) ≥7.0 retained for downstream processing. cDNA libraries were prepared using the TruSeq Stranded mRNA Library Prep Kit (Illumina, Inc.) according to the manufacturer’s protocol. Briefly, polyadenylated mRNA was enriched from 1 µg of total RNA using oligo(dT) magnetic beads and fragmented (200–600 bp) via divalent cation-mediated cleavage at 85 °C for 6 minutes. First- and second-strand cDNA synthesis was performed using the fragmented mRNA as a template. To ensure strand specificity, dUTP was incorporated during second-strand synthesis, followed by enzymatic degradation of the dUTP-containing strand using uracil DNA glycosylase. The resulting cDNA fragments were end-repaired, A-tailed, and ligated to adapters. After purification, the first-strand cDNA was PCR-amplified (12–15 cycles) to generate sequencing-ready libraries. Library quality was verified using an Agilent 2200 TapeStation system, followed by paired-end (150 bp) sequencing on a DNBSEQ-T7 platform (MGI Tech Co., Ltd.) to obtain approximately 20 million reads per sample.

Raw sequencing reads were quality-checked using FastQC (v0.11.9) and trimmed for adapters and low-quality bases using Trim Galore (v0.6.7). Reads were aligned to the [reference genome, e.g., hg38] using STAR (v2.7.10a) with default parameters. Gene expression was quantified using featureCounts (v2.0.1), and differential gene expression analysis was performed with DESeq2 (v1.34.0). Functional enrichment of differentially expressed genes was evaluated using Gene Ontology (GO) and Kyoto Encyclopedia of Genes and Genomes (KEGG) pathway analyses via clusterProfiler (v4.2.2).

### RNA extraction and quantitative Real-Time PCR (qRT-PCR)

Total RNA was extracted using the EZBioscience RNA Extraction Kit according to the manufacturer’s protocol. cDNA was synthesized from 1 µg of total RNA using the Reverse Transcription Kit following the supplier’s instructions. qRT-PCR was performed using the Vazyme SYBR Green Master Mix on a QuantStudio Dx instrument. Each reaction was run in triplicate, and gene expression levels were quantified using the 2−ΔΔCt method, with ACTB as the internal reference gene. A total of three independent biological replicates were performed. Primer sequences, designed and synthesized by Sangon Biotech (Shanghai, China), are listed in the Supplementary Table 2.

### Dual Luciferase reporter gene assay

The 2.1 kb promoter region of GFPT2 (−2000 to +100 bp relative to the transcription start site) was amplified by PCR and cloned into the pGL3 luciferase reporter vector (Sangon Biotech, Shanghai, China). Cells were co-transfected with the pGL3-NSUN2 reporter construct and the pRL-TK normalization plasmid (Sangon Biotech, Shanghai, China) using Lipofectamine 3000. Luciferase activity was measured 48 hours post-transfection using the Dual-Luciferase Reporter Assay System according to the manufacturer’s protocol. A total of three independent biological replicates were performed. Firefly luciferase activity was normalized to Renilla luciferase activity, and data were analyzed as relative luciferase units (RLU).

### Co-Immunoprecipitation (Co-IP)

Proteins were extracted using NP-40 lysis buffer supplemented with a protease and phosphatase inhibitor cocktail, and obtained protein lysates were then incubated with the corresponding antibodies and magnetic beads overnight at 4 °C. The immunoprecipitated complexes were subjected to the immunoblotting assays.

### UDP-GlcNAc measurement

UDP-GlcNAc levels were measured using a commercially available UDP-GlcNAc assay kit, following the protocols provided by the manufacturer. A total of three independent biological replicates were performed.

### Seahorse assay, glucose uptake, and lactate production

A Seahorse XF96 Extracellular Flux Analyzer (Seahorse Bioscience Inc., North Billerica, MA, USA) was used to assess glycolytic activity and mitochondrial function by measuring the extracellular acidification rate (ECAR), according to the manufacturer’s instructions. Glucose uptake and lactate production were measured using commercially available glucose and lactate assay kits, following the respective protocols provided by the manufacturer.

### Structural analysis

The crystal structure of human Raptor-Rags complex with accession code 6U62 was downloaded from the Protein Data Bank (PDB). The figure was generated using PyMOL. Multiple sequence alignment of Raptor amino acid sequences from different species was generated using MEGA.

### Public datasets analysis

Gene expression levels of LBX2 in tumor and normal tissues from CRC patients were analyzed in the The Cancer Genome Atlas (TCGA) database using the GEPIA2 platform (http://gepia2.cancer-pku.cn/#index) or in Gene Expression Omnibus (GEO) datasets. Based on the median LBX2 expression, CRC patients were stratified into LBX2 high-expression and low-expression groups. To evaluate the prognostic significance of LBX2 expression, Kaplan-Meier survival curves were generated for each group. Differences in survival between the high and low expression groups were assessed using the log-rank test. Spearman correlation analysis was performed to assess the expression levels of LBX2 and GFPT2. Potential LBX2 binding sites within the GFPT2 promoter region (−2000 to +100 bp) were identified using the JASPAR database based on the LBX2 binding motif.

### Statistical analysis

Continuous variables were analyzed using Student’s t-test or the Wilcoxon rank-sum test for comparisons between two groups, depending on data distribution, and one-way analysis of variance (ANOVA) or the Kruskal–Wallis test for comparisons among multiple groups. Categorical variables were evaluated using the chi-square test or Fisher’s exact test, as appropriate. Overall survival was estimated with the Kaplan–Meier method, and differences between survival curves were assessed using the log-rank test. All statistical analyses were conducted using GraphPad Prism v9.0 (GraphPad Software, La Jolla, CA, USA) and R v4.1.0 (CRAN, https://cran.r-project.org). A two-sided P-value < 0.05 was considered statistically significant unless otherwise specified.

## Supplementary information

Supplementary Figures

Supplementary Table 1

Supplementary Table 2

Supplementary Table 3

Orginal Western Blot Figures

Supplementary Table 4

Supplementary Table 5

## Data Availability

The raw sequence data reported in this paper have been deposited in the Genome Sequence Archive (Genomics, Proteomics & Bioinformatics 2025) in the National Genomics Data Center (Nucleic Acids Res 2025), China National Center for Bioinformation / Beijing Institute of Genomics, Chinese Academy of Sciences (GSA-Human: HRA013742) which are publicly accessible at https://ngdc.cncb.ac.cn/gsa-human/browse/HRA013742.

## References

[1] Sung H, Ferlay J, Siegel RL, Laversanne M, Soerjomataram I, Jemal A, et al. Global Cancer Statistics 2020: GLOBOCAN Estimates of Incidence and Mortality Worldwide for 36 Cancers in 185 Countries. CA Cancer J Clin. 2021;71:209–49. 33538338 10.3322/caac.21660

[2] Dekker E, Tanis PJ, Vleugels JLA, Kasi PM, Wallace MB. Colorectal cancer. Lancet. 2019;394:1467–80. 31631858 10.1016/S0140-6736(19)32319-0

[3] Abedizadeh R, Majidi F, Khorasani HR, Abedi H, Sabour D. Colorectal cancer: a comprehensive review of carcinogenesis, diagnosis, and novel strategies for classified treatments. Cancer Metastasis Rev. 2024;43:729–53. 38112903 10.1007/s10555-023-10158-3

[4] Li J, Ma X, Chakravarti D, Shalapour S, DePinho RA. Genetic and biological hallmarks of colorectal cancer. Genes Dev. 2021;35:787–820. 34074695 10.1101/gad.348226.120 PMC8168558

[5] Moisan V, Bomgardner D, Tremblay JJ. Expression of the Ladybird-like homeobox 2 transcription factor in the developing mouse testis and epididymis. BMC Dev Biol. 2008;8:22. 18304314 10.1186/1471-213X-8-22 PMC2277406

[6] Moisan V, Robert NM, Tremblay JJ. Expression of ladybird-like homeobox 2 (LBX2) during ovarian development and folliculogenesis in the mouse. J Mol Histol. 2010;41:289–94. 20820887 10.1007/s10735-010-9291-5

[7] Ochi H, Westerfield M. Lbx2 regulates formation of myofibrils. BMC Dev Biol. 2009;9:13. 19216761 10.1186/1471-213X-9-13 PMC2656488

[8] Lou Q, He J, Hu L, Yin Z. Role of lbx2 in the noncanonical Wnt signaling pathway for convergence and extension movements and hypaxial myogenesis in zebrafish. Biochim Biophys Acta. 2012;1823:1024–32. 22406073 10.1016/j.bbamcr.2012.02.013

[9] Lu FI, Sun YH, Wei CY, Thisse C, Thisse B. Tissue-specific derepression of TCF/LEF controls the activity of the Wnt/β-catenin pathway. Nat Commun. 2014;5:5368. 25371059 10.1038/ncomms6368

[10] Wang J, Luo J, Chen Q, Wang X, He J, Zhang W, et al. Identification of LBX2 as a novel causal gene of atrial septal defect. Int J Cardiol. 2018;265:188–94. 29669692 10.1016/j.ijcard.2018.04.038

[11] Huang X, Yang Y, Yang C, Li H, Cheng H, Zheng Y. Overexpression of LBX2 associated with tumor progression and poor prognosis in colorectal cancer. Oncol Lett. 2020;19:3751–60. 32382328 10.3892/ol.2020.11489 PMC7202318

[12] Hu J, Bai Y, Zhang Q, Li M, Yin R, Xu L. Identification of LBX2 as a novel causal gene of lung adenocarcinoma. Thorac Cancer. 2020;11:2137–45. 32567804 10.1111/1759-7714.13506 PMC7396393

[13] Xiong J, Liang H, Sun X, Gao K. Histone modification-linked prognostic model for ovarian cancer reveals LBX2 as a novel growth promoter. J Cell Mol Med. 2024;28:e18260. 38520216 10.1111/jcmm.18260 PMC10960176

[14] Eichler J. Protein glycosylation. Curr Biol. 2019;29:R229–r31. 30939300 10.1016/j.cub.2019.01.003

[15] He XF, Hu X, Wen GJ, Wang Z, Lin WJ. O-GlcNAcylation in cancer development and immunotherapy. Cancer Lett. 2023;566:216258. 37279852 10.1016/j.canlet.2023.216258

[16] Dupas T, Lauzier B, McGraw S. O-GlcNAcylation: the sweet side of epigenetics. Epigenet Chromatin. 2023;16:49. 10.1186/s13072-023-00523-5 PMC10720106 38093337

[17] Paneque A, Fortus H, Zheng J, Werlen G, Jacinto E. The Hexosamine Biosynthesis Pathway: Regulation and Function. Genes (Basel). 2023;14. 10.3390/genes14040933 PMC10138107 37107691

[18] Lam C, Low JY, Tran PT, Wang H. The hexosamine biosynthetic pathway and cancer: Current knowledge and future therapeutic strategies. Cancer Lett. 2021;503:11–8. 33484754 10.1016/j.canlet.2021.01.010

[19] Pinho SS, Reis CA. Glycosylation in cancer: mechanisms and clinical implications. Nat Rev Cancer. 2015;15:540–55. 26289314 10.1038/nrc3982

[20] Xu X, Peng Q, Jiang X, Tan S, Yang W, Han Y, et al. Altered glycosylation in cancer: molecular functions and therapeutic potential. Cancer Commun. 2024;44:1316–36. 10.1002/cac2.12610 PMC11570773 39305520

[21] Nie H, Ju H, Fan J, Shi X, Cheng Y, Cang X, et al. O-GlcNAcylation of PGK1 coordinates glycolysis and TCA cycle to promote tumor growth. Nat Commun. 2020;11:36. 31911580 10.1038/s41467-019-13601-8 PMC6946671

[22] He X, Li Y, Chen Q, Zheng L, Lou J, Lin C, et al. O-GlcNAcylation and stablization of SIRT7 promote pancreatic cancer progression by blocking the SIRT7-REGγ interaction. Cell Death Differ. 2022;29:1970–81. 35422493 10.1038/s41418-022-00984-3 PMC9525610

[23] Ayodeji SA, Bao B, Teslow EA, Polin LA, Dyson G, Bollig-Fischer A, et al. Hyperglycemia and O-GlcNAc transferase activity drive a cancer stem cell pathway in triple-negative breast cancer. Cancer Cell Int. 2023;23:102. 37231419 10.1186/s12935-023-02942-6 PMC10210312

[24] Le Minh G, Merzy J, Esquea EM, Ahmed NN, Young RG, Sharp RJ, et al. GATAD2B O-GlcNAcylation regulates breast cancer stem-like potential and drug resistance. Cells. 2025;14. 10.3390/cells14060398 PMC11941746 40136647

[25] Liu YY, Liu HY, Yu TJ, Lu Q, Zhang FL, Liu GY, et al. O-GlcNAcylation of MORC2 at threonine 556 by OGT couples TGF-β signaling to breast cancer progression. Cell Death Differ. 2022;29:861–73. 34974534 10.1038/s41418-021-00901-0 PMC8991186

[26] Chen Y, Bei J, Liu M, Huang J, Xie L, Huang W, et al. Sublethal heat stress-induced O-GlcNAcylation coordinates the Warburg effect to promote hepatocellular carcinoma recurrence and metastasis after thermal ablation. Cancer Lett. 2021;518:23–34. 34126196 10.1016/j.canlet.2021.06.001

[27] Xu C, Pan X, Wang D, Guan Y, Yang W, Chen X, et al. O-GlcNAcylation of Raptor transduces glucose signals to mTORC1. Mol Cell. 2023;83:3027–40.e11. 37541260 10.1016/j.molcel.2023.07.011

[28] Miszkiel A, Wojciechowski M. Long range molecular dynamics study of interactions of the eukaryotic glucosamine-6-phosphate synthase with fructose-6-phosphate and UDP-GlcNAc. J Mol Graph Model. 2017;78:14–25. 28968565 10.1016/j.jmgm.2017.09.009

[29] Yang Z, Dong X, Pu M, Yang H, Chang W, Ji F, et al. LBX2-AS1/miR-219a-2-3p/FUS/LBX2 positive feedback loop contributes to the proliferation of gastric cancer. Gastric Cancer. 2020;23:449–63. 31673844 10.1007/s10120-019-01019-6

[30] Balasubramani A, Rao A. O-GlcNAcylation and 5-methylcytosine oxidation: an unexpected association between OGT and TETs. Mol Cell. 2013;49:618–9. 23438858 10.1016/j.molcel.2013.02.006 PMC3770526

[31] Goul C, Peruzzo R, Zoncu R. The molecular basis of nutrient sensing and signalling by mTORC1 in metabolism regulation and disease. Nat Rev Mol Cell Biol. 2023;24:857–75. 37612414 10.1038/s41580-023-00641-8

[32] Liu X, Yamaguchi K, Takane K, Zhu C, Hirata M, Hikiba Y, et al. Cancer-associated IDH mutations induce Glut1 expression and glucose metabolic disorders through a PI3K/Akt/mTORC1-Hif1α axis. PLoS One. 2021;16:e0257090. 34516556 10.1371/journal.pone.0257090 PMC8437293

[33] Lv X, Lv Y, Dai X. Lactate, histone lactylation and cancer hallmarks. Expert Rev Mol Med. 2023;25:e7. 36621008 10.1017/erm.2022.42

[34] Chen J, Huang Z, Chen Y, Tian H, Chai P, Shen Y, et al. Lactate and lactylation in cancer. Signal Transduct Target Ther. 2025;10:38. 39934144 10.1038/s41392-024-02082-x PMC11814237

[35] Chen L, Huang L, Gu Y, Cang W, Sun P, Xiang Y. Lactate-Lactylation hands between metabolic reprogramming and immunosuppression. Int J Mol Sci. 2022;23. 10.3390/ijms231911943 PMC9569569 36233246

[36] Yang J, Yu X, Xiao M, Xu H, Tan Z, Lei Y, et al. Histone lactylation-driven feedback loop modulates cholesterol-linked immunosuppression in pancreatic cancer. Gut. 2025. 10.1136/gutjnl-2024-334361 PMC12573332 40467104

[37] Lu B, Chen S, Guan X, Chen X, Du Y, Yuan J, et al. Lactate accumulation induces H4K12la to activate super-enhancer-driven RAD23A expression and promote niraparib resistance in ovarian cancer. Mol Cancer. 2025;24:83. 40102876 10.1186/s12943-025-02295-w PMC11921584

[38] Poeppel TD, Krause BJ, Heusner TA, Boy C, Bockisch A, Antoch G. PET/CT for the staging and follow-up of patients with malignancies. Eur J Radio. 2009;70:382–92. 10.1016/j.ejrad.2009.03.051 19406595

[39] Driehuis E, Kretzschmar K, Clevers H. Establishment of patient-derived cancer organoids for drug-screening applications. Nat Protoc. 2020;15:3380–409. 32929210 10.1038/s41596-020-0379-4

[40] Grönholm M, Feodoroff M, Antignani G, Martins B, Hamdan F, Cerullo V. Patient-derived organoids for precision cancer immunotherapy. Cancer Res. 2021;81:3149–55. 33687948 10.1158/0008-5472.CAN-20-4026 PMC7616950

[41] Wang L, Zhuang B, Jiang Y, Chen Z, Ge C, Yu M, et al. Hypoxia-induced HIF1A impairs sorafenib sensitivity in hepatocellular carcinoma through NSUN2-mediated stabilization of GDF15. Cell Signal. 2025;135:112076. 40840765 10.1016/j.cellsig.2025.112076

