# Supplementary material for: LBX2 promotes colorectal cancer progression via the glycosylation and lactylation positive feedback
Source: Cell Death Discov. 2025 Dec 12;11:556. doi: 10.1038/s41420-025-02888-w (PMC12700955; doi:10.1038/s41420-025-02888-w)
Supplement: Supplementary file 1 — Supplementary Figures [file 41420_2025_2888_MOESM1_ESM.docx]

**LBX2 Promotes Colorectal Cancer Progression via the Glycosylation and Lactylation Positive Feedback**

**Authors and Affiliations**

Author names:

Yiwen Jiang^1,2^, Lude Wang^3,4^, Lin Chen^3,4^, Kai Shen^2^, Jie Chang^3,4^, Shicong Zheng^1^, Zewei Chen^1^, Chenyang Ge^1^, Min Yu^1^, Shian Yu^1^, Haiping Lin^1^

Author affiliations:

1. Department of General Surgery, Jinhua Central Hospital, Teaching Hospital of Mathematical Medicine College, Zhejiang Normal University, Zhejiang, China

2. Department of Blood Transfusion, Affiliated Jinhua Hospital, Zhejiang University School of Medicine, Zhejiang, China

3. Key Laboratory of Nutrition and Metabolism Research for Oncology, Affiliated Jinhua Hospital, Zhejiang University School of Medicine, Zhejiang, China

4. Central Laboratory and Precision Medicine Center, Affiliated Jinhua Hospital, Zhejiang University School of Medicine, Zhejiang, China

# Yiwen Jiang, Lude Wang and Lin Chen contribute equally to the work and should be considered co-first authors.

*Author for correspondence:

Haiping Lin (E-mail: lhp1994@sjtu.edu.cn)

Shian Yu (E-mail: zjjhysa@163.com)

Min Yu (E-mail: greendoctor@163.com)

**Subject terms:** Colorectal cancer; LBX2; O-GlcNAcylation; Histone lactylation; Glycolysis

**Supplementary Figures**

**
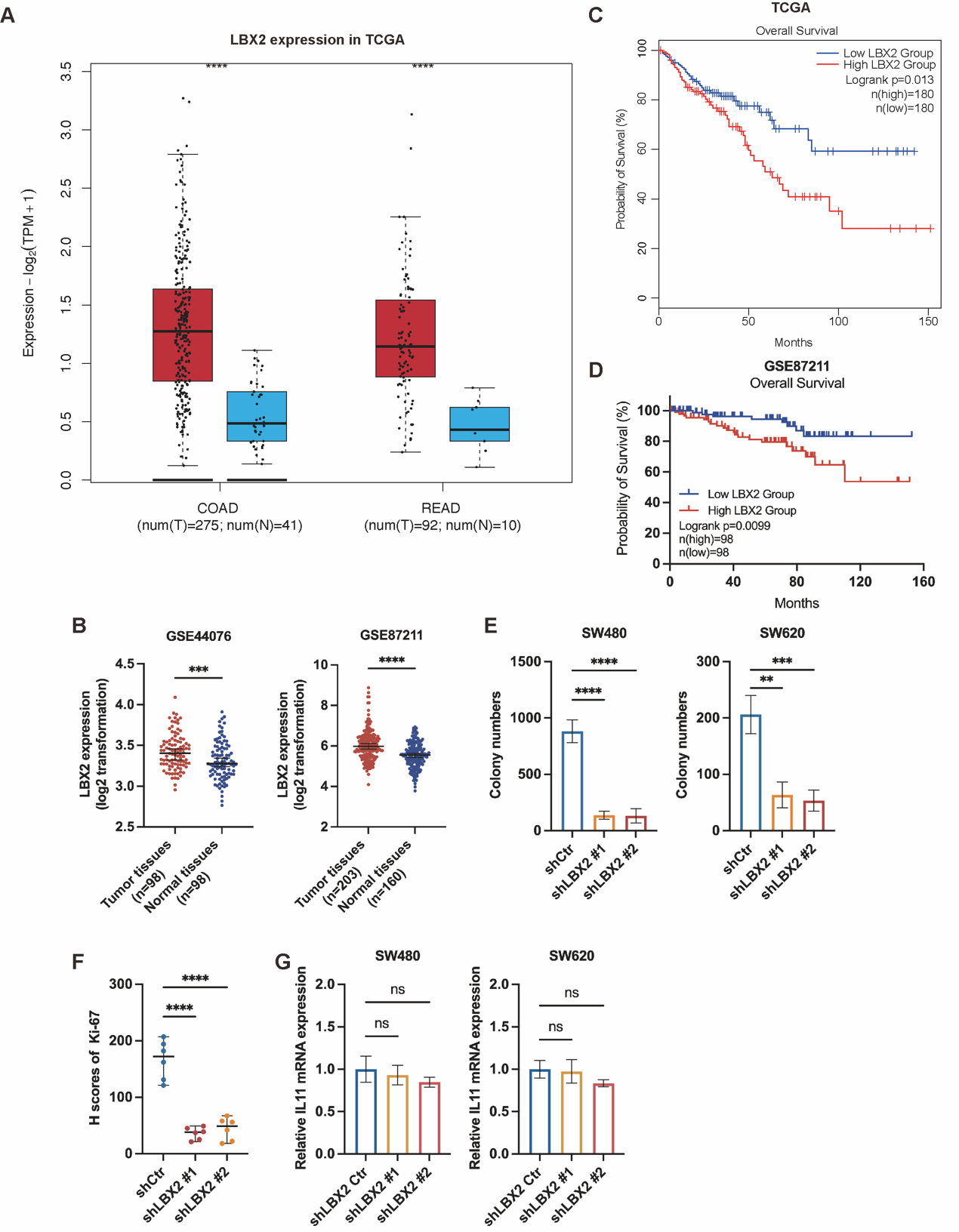
**

**Supplementary Figure 1.** (A, B) Expression of LBX2 between tumor tissues and normal tissues in colon adenocarcinoma (COAD) and rectal adenocarcinoma (READ) from the TCGA database and the GEO database (GSE44076 and GSE87211); (C, D) CRC patients from the TCGA database and the GEO database (GSE87211) were stratified into LBX2 high-expression and low-expression groups based on cohort-specific median LBX2 expression levels in tumor tissues. Kaplan-Meier survival analysis and log-rank test were performed to compare overall survival (OS) outcomes between the two groups; (E) Quantification of colony formation in SW480 and SW620 cells following LBX2 knockdown; (F) IHC H-scores of Ki-67 in mouse tumor tissues comparing the LBX2 knockdown group with the control group. (G) qRT-PCR analysis of IL11 mRNA expression levels in SW480 and SW620 cells transfected with shCtr, shLBX2 #1, and shLBX2 #2. ** P < 0.01; *** P < 0.001; **** P < 0.0001.


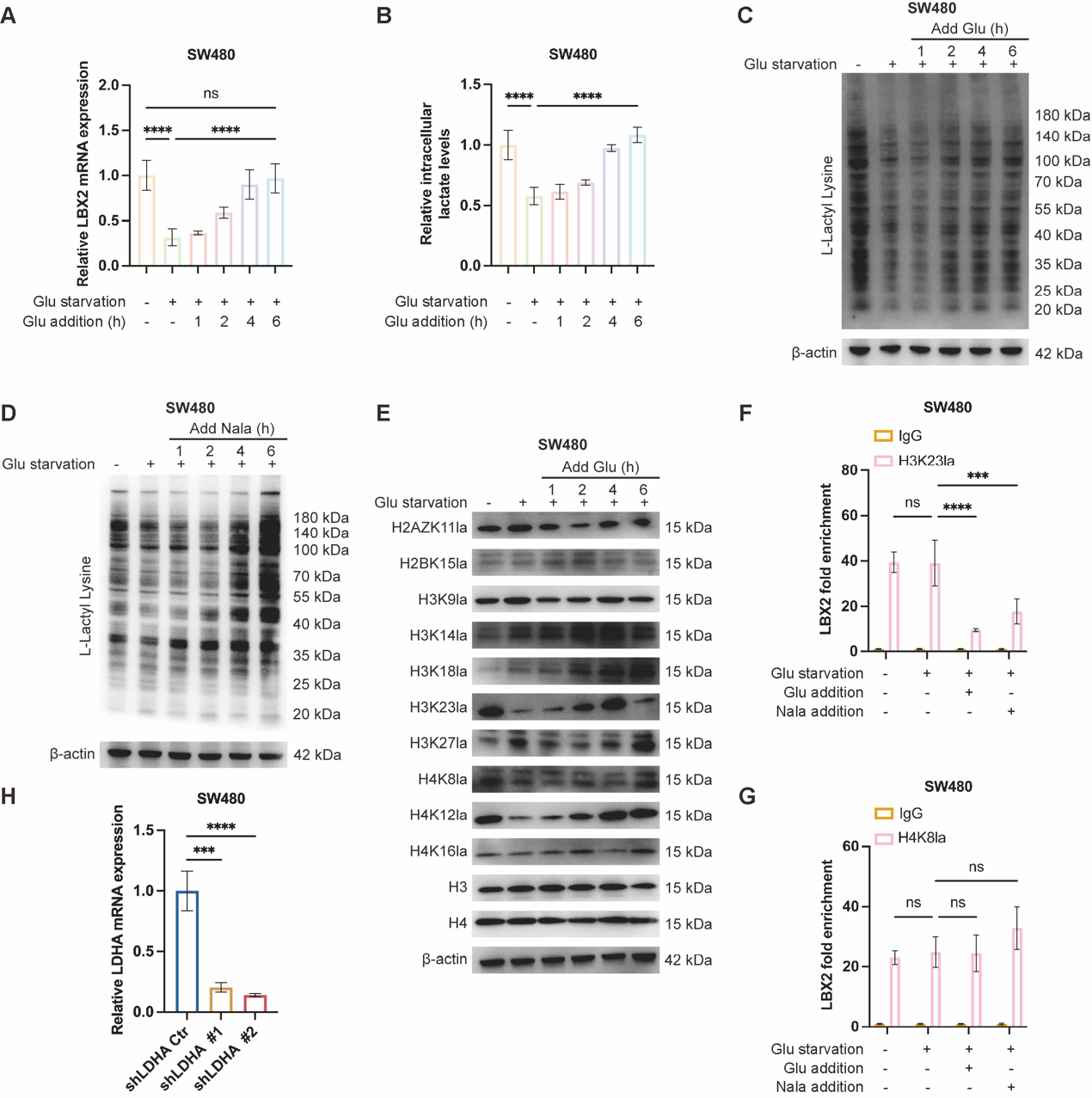


**Supplementary Figure 2.** (A) qRT-PCR analysis of LBX2 mRNA expression levels in SW480 cells under glucose restriction (1.0 mM for 12 h) and exogenous glucose (25.0 mM for 6 h) supplementation; (B) ELISA analysis of intracellular lactate levels in SW480 cells under glucose restriction (1.0 mM for 12 h) and exogenous glucose supplementation (25.0 mM) for a specified duration; (C, D) Western blot analysis of total lactylation levels in SW480 cells under glucose restriction (1.0 mM for 12 h) and exogenous glucose (25.0 mM for 6 h) (C) or sodium lactate (5.0 mM for 6 h) (D) supplementation; (E) Western blot analysis of site-specific histone lactylation in SW480 cells under glucose restriction (1.0 mM for 12 h) and exogenous glucose supplementation (25.0 mM for 6 h); (F, G) ChIP-qPCR analysis of H3K23la or H4K8la enrichment at the LBX2 promoter region in SW480 cells under glucose deprivation (1.0 mM for 12 h) and glucose (25.0 mM for 6 h) or sodium lactate (5.0 mM for 6 h) supplementation. (H) qRT-PCR analysis to assess LDHA knockdown efficiency in SW480 cell with stable LDHA knockdown. *** P < 0.001; **** P < 0.0001.
