# Supplementary material for: LBX2 promotes colorectal cancer progression via the glycosylation and lactylation positive feedback
Source: Cell Death Discov. 2025 Dec 12;11:556. doi: 10.1038/s41420-025-02888-w (PMC12700955; doi:10.1038/s41420-025-02888-w)
Supplement: Supplementary file 2 — Supplementary Table 1 [file 41420_2025_2888_MOESM2_ESM.docx]

| **Supplementary Table 1. Oligonucleotides，recombinant DNA and software used in the study** | | |
| --- | --- | --- |
| **REAGENT or RESOURCE** | **SOURCE** | **IDENTIFIER** |
| **Oligonucleotides** | | |
| LBX2 shRNA_1 | GCGCTTCGTCTTCCAGAAGTA | N/A |
| LBX2 shRNA_2 | TGGTCACTTGGTTCCAGAACC | N/A |
| GFPT2 shRNA_1 | ACCATCGCCAAGCTGATTAAA | N/A |
| GFPT2 shRNA_2 | AGGTAACTTCAGTGCGTTTAT | N/A |
| LDHA shRNA_1 | CCACCATGATTAAGGGTCTTT | N/A |
| LDHA shRNA_2 | CCAAAGATTGTCTCTGGCAAA | N/A |
| RT-PCR primer-LBX2 | CACTCTTAAGCATCGCAGACA CTTCTGGAAGACGAAGCGCC | N/A |
| RT-PCR primer-GFPT2 | CCAGGGTCGCCCCATTATAC TGGTGATGGTCTTGTCACGG | N/A |
| RT-PCR primer-LDHA | ACGTGCATTCCCGATTCCTT AAAGGCTGCCATGTTGGAGA | N/A |
| RT-PCR primer-IL11 | ATGAACTGTGTTTGCCGCCT GGAATCCAGGTTGTGGTCCC | N/A |
| RT-PCR primer-ACTIN | GCGAGCACACGAGCCTCGCCTT CATCATCCATGGTGAGCTGGCGG | N/A |
| ChIP-qPCR primer-GFPT2 | CAGCTTCGTTCACAACCTGC TGGCGGAGAGACGAGAAAAC | N/A |
| ChIP-qPCR primer-GAPDH | TGCTGAGTCACCTTCGAACC ACTGTCTTCTCCCCGCAAAG | N/A |
| ChIP-qPCR primer-LBX2 | ACACTCATGCTGTGTCCTGC GGAGGCCTGGTTTCACACATA | N/A |
| **Recombinant DNA** | | |
| pLenti-Flag-WT | This paper | N/A |
| pLenti-Flag-LBX2 | This paper | N/A |
| pCDNA3.1-WT | This paper | N/A |
| pCDNA3.1-LBX2 | This paper | N/A |
|  | This paper | N/A |
| pCDNA3.1-Raptor | This paper | N/A |
| pCDNA3.1-Raptor (T700A) | This paper | N/A |
| pRL-TK | This paper | N/A |
| pGL3-Basic-Luc | This paper | N/A |
| pGL3-GFPT2 WT-Luc | This paper | N/A |
| pGL3-GFPT2 (-400— +100)-Luc | This paper | N/A |
| pGL3-GFPT2 (-800— -401; +1—+100)-Luc | This paper | N/A |
| pGL3-GFPT2 (-2000— -801; +1—+100)-Luc | This paper | N/A |
| pGL3-GFPT2 LBE1-Luc | This paper | N/A |
| pGL3-GFPT2 LBE2-Luc | This paper | N/A |
| pGL3-GFPT2 LBE3-Luc | This paper | N/A |
| **Software and algorithms** | | |
| SPSS 22.0 | IBM | https://www.ibm.com/cn-zh/spss |
| R 4.4.2 | N/A | https://cran.r-project.org/ |
| Prism 8.0 | GraphPad | https://www.graphpad.com/ |
| ImageJ | N/A | https://imagej.nih.gov/ij/ |
| FlowJo | N/A | https://www.flowjo.com/ |
| AlphaFold3 | N/A | https://alphafold.ebi.ac.uk/ |
| PyMOL IBM | N/A | https://www.pymol.org/ |
