# Supplementary material for: LBX2 promotes colorectal cancer progression via the glycosylation and lactylation positive feedback
Source: Cell Death Discov. 2025 Dec 12;11:556. doi: 10.1038/s41420-025-02888-w (PMC12700955; doi:10.1038/s41420-025-02888-w)
Supplement: Supplementary file 3 — Supplementary Table 2 [file 41420_2025_2888_MOESM3_ESM.docx]

| **Supplementary Table 2. Reagent and resources used in the study** | | |
| --- | --- | --- |
| **REAGENT or RESOURCE** | **SOURCE** | **IDENTIFIER** |
| **Antibodies** | | |
| LBX2 antibody (WB) | Thermo Fisher Scientific | Cat# PA5-69480 |
| LBX2 antibody (IHC) | Bioss | Cat# bs-17114R |
| ki-67 | Cell Signaling Technology | Cat# 9449 |
| GFPT2 antibody | Proteintech | Cat# 15189-1-AP |
| O-linked N-acetylglucosamine (O-GlcNAc) antibody | Thermo Fisher Scientific | Cat# MA1-072 |
| Akt antibody | Cell Signaling Technology | Cat# 9272 |
| Phospho-Akt antibody | Cell Signaling Technology | Cat# 4060 |
| mTOR antibody | Cell Signaling Technology | Cat# 2983 |
| Phospho-mTOR antibody | Cell Signaling Technology | Cat# 5536 |
| p70 S6 Kinase antibody | Cell Signaling Technology | Cat# 2708 |
| Phospho-p70 S6 Kinase antibody | Cell Signaling Technology | Cat# 9234 |
| Rag A/B | Santa Cruz Biotechnology | Cat# 41386 |
| L-Lactyl Lysine antibody | PTMBIO | Cat# PTM-1401RM |
| L-Lactyl-Histone Antibody Sampler Kit | PTMBIO | Cat# PTM-7483 |
| β-Actin antibody | Beyotime | Cat# AF5003 |
| Flag antibody | Proteintech | Cat# 20543-1-AP |
| HA antibody | Proteintech | Cat# 51064-2-AP |
| **Bacterial and virus strains** | | |
| Competent E.coli DH5a | WEIDI Biosciences | Cat# DL1001M |
| **Biological samples** | | |
| Tissues from CRC patients | Jinhua Municipal Central Hospital | N/A |
| **Chemicals, peptides, and recombinant proteins** | | |
| Protease inhibitor cocktail | Selleck | Cat# B14001 |
| Phosphatase inhibitor cocktail | Selleck | Cat# B15001 |
| DAPI | Beyotime | Cat# C1341S |
| Lipofectamine 3000 | Thermo Fisher Scientific | Cat# L3000015 |
| Puromycin | Yeasen | Cat# 60210ES25 |
| UDP-GlcNAc disodium | MedChemExpress | Cat# HY-112174 |
| OGT-IN-1 | MedChemExpress | Cat# HY-101548 |
| Lactate sodium | MedChemExpress | Cat# HY-B2227B |
| 2-Deoxy-D-glucose | MedChemExpress | Cat# HY-13966 |
| (Human) Colorectal cancer organoid culture medium | OmaStem | Cat# OM11 |
| **Critical commercial assays** | | |
| Dual Luciferase Reporter Assay Kit | Vazyme | Cat# DL101-01 |
| SimpleChIP® Plus Enzymatic Chromatin IP Kit (Magnetic Beads) | Cell Signaling Technology | Cat# 9005 |
| CCK-8 Cell Counting Kit | Vazyme | Cat# A311-01 |
| Human UDP-GlcNAc ELISA kit | BiotechPack | Cat# EKHU2927 |
| L-Lactate Assay Kit with WST-8 | Beyotime | Cat# S0208S |
| Seahorse XF Cell Glycosis Stress Test Kit | Agilent | Cat# 103020-100 |
| Protein A+G Magnetic Beads | Beyotime | Cat# P2108-5ml |
| cDNA Synthesis Kit | YEASEN | Cat# 11121ES60 |
| SYBR Green Master Mix | YEASEN | Cat# 11202ES60 |
| CellTiter-Blue® Cell Viability Assay | Promega | Cat# G8080 |
| **Experimental models** | | |
| BALB/c nude mice | GemPharmatech | N/A |
