# Supplementary material for: LBX2 promotes colorectal cancer progression via the glycosylation and lactylation positive feedback
Source: Cell Death Discov. 2025 Dec 12;11:556. doi: 10.1038/s41420-025-02888-w (PMC12700955; doi:10.1038/s41420-025-02888-w)
Supplement: Supplementary file 4 — Supplementary Table 3 [file 41420_2025_2888_MOESM4_ESM.docx]

**Supplementary Table 3. Patient demographics and clinicopathological characteristics of the JHYY cohort**

| **Characteristic** | **Overall  N = 50** | **High LBX2 Group  N = 25** | **Low LBX2 Group  N = 25** | **P value** |
| --- | --- | --- | --- | --- |
| **Age (Mean ± SD)** | 64 ± 11 | 66 ± 11 | 63 ± 10 | 0.277^1^ |
| **Gender, n (%)** |  |  |  | 0.382^2^ |
| Male | 31 (62.00%) | 14 (56.00%) | 17 (68.00%) |  |
| Female | 19 (38.00%) | 11 (44.00%) | 8 (32.00%) |  |
| **Tumor Location, n (%)** |  |  |  | 0.777^2^ |
| Colon | 25 (50.00%) | 12 (48.00%) | 13 (52.00%) |  |
| Rectal | 25 (50.00%) | 13 (52.00%) | 12 (48.00%) |  |
| **Maximum Tumor Diameter (Mean ± SD)** | 4.79 ± 1.87 | 5.63 ± 1.62 | 3.95 ± 1.75 | <0.001^1^ |
| **Grade (%)** |  |  |  | 0.013^2^ |
| G1 | 18 (36.00%) | 4 (16.00%) | 14 (56.00%) |  |
| G2 | 20 (40.00%) | 13 (52.00%) | 7 (28.00%) |  |
| G3 | 12 (24.00%) | 8 (32.00%) | 4 (16.00%) |  |
| **T Stage (%)** |  |  |  | 0.003^3^ |
| T1 | 1 (2.00%) | 0 (0.00%) | 1 (4.00%) |  |
| T2 | 13 (26.00%) | 3 (12.00%) | 10 (40.00%) |  |
| T3 | 29 (58.00%) | 15 (60.00%) | 14 (56.00%) |  |
| T4 | 7 (14.00%) | 7 (28.00%) | 0 (0.00%) |  |
| **N Stage (%)** |  |  |  | 0.084^3^ |
| N0 | 22 (44.00%) | 8 (32.00%) | 14 (56.00%) |  |
| N1 | 21 (42.00%) | 11 (44.00%) | 10 (40.00%) |  |
| N2 | 7 (14.00%) | 6 (24.00%) | 1 (4.00%) |  |
| **M Stage (%)** |  |  |  | 0.765^2^ |
| M0 | 33 (66.00%) | 17 (68.00%) | 16 (64.00%) |  |
| M1 | 17 (34.00%) | 8 (32.00%) | 9 (36.00%) |  |
| ^1^Welch Two Sample t-test | | | | |
| ^2^Pearson's Chi-squared test | | | | |
| ^3^Fisher's exact test | | | | |
