# Supplementary material for: LBX2 promotes colorectal cancer progression via the glycosylation and lactylation positive feedback
Source: Cell Death Discov. 2025 Dec 12;11:556. doi: 10.1038/s41420-025-02888-w (PMC12700955; doi:10.1038/s41420-025-02888-w)

Figure 1

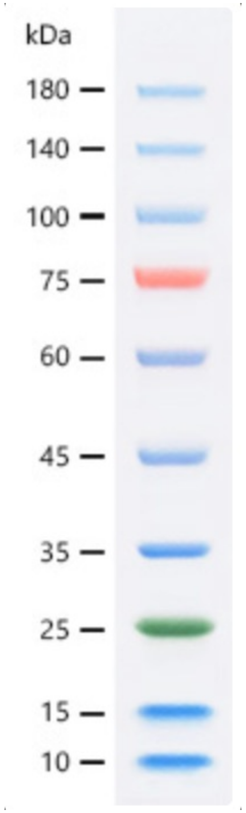

Western blot marker used in the study
(Abclonal Cat# RM19001)

LBX2

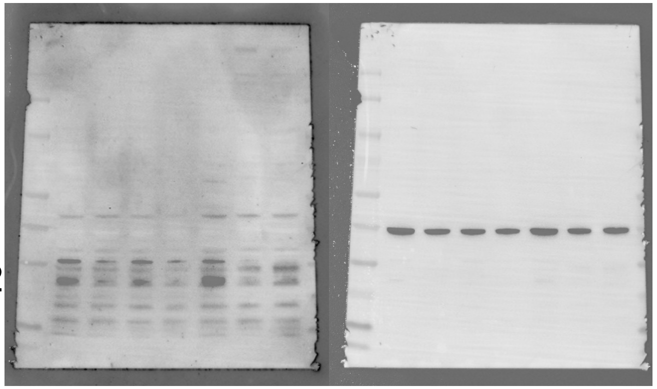

β-Actin

Figure 1D

LBX2

β-Actin

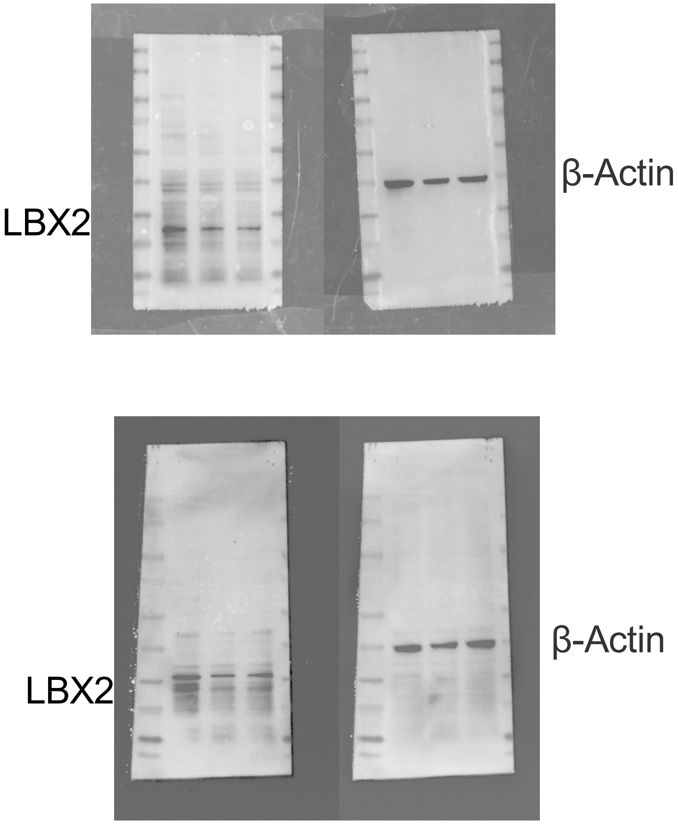

LBX2

β-Actin

Figure 1E

Figure 3

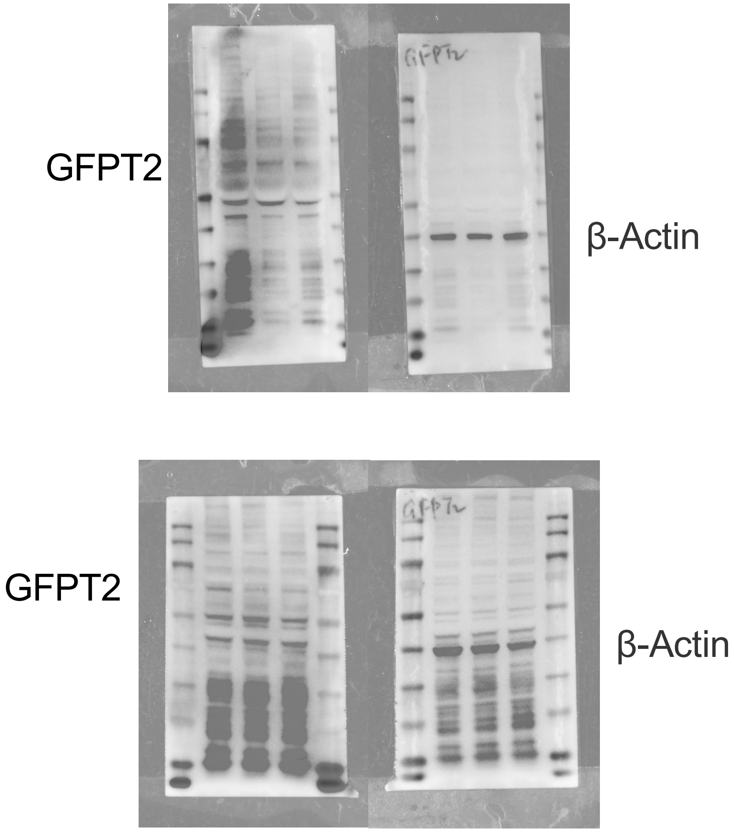

Figure 3B

Figure 4

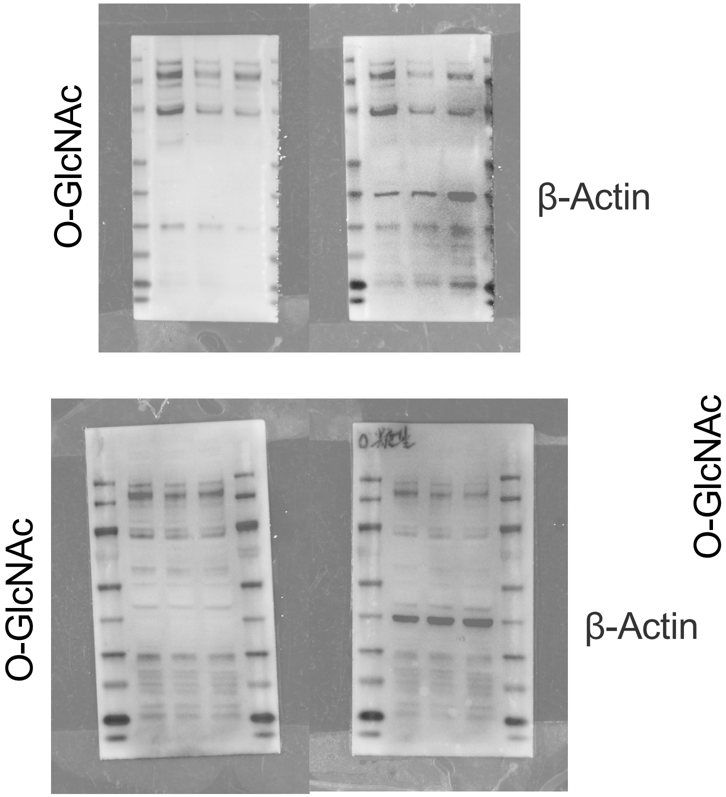

Figure 4C

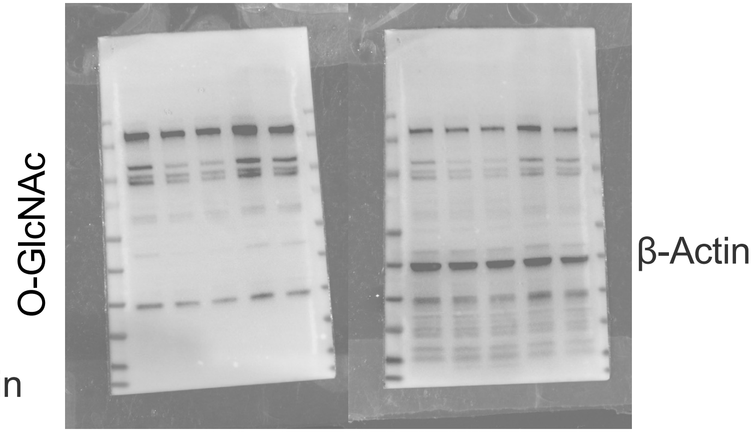

Figure 4E

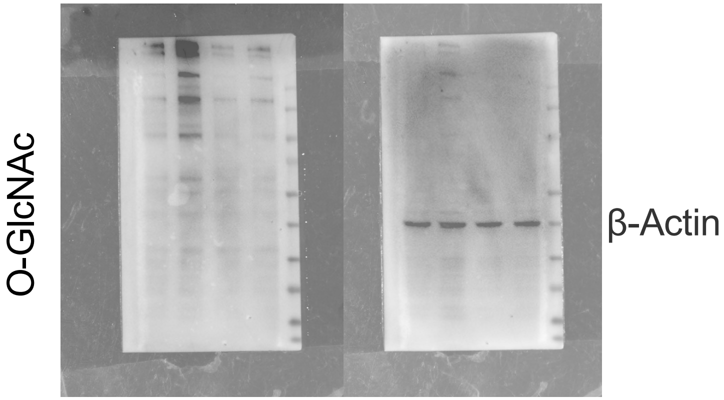

Figure 4F

Figure 5

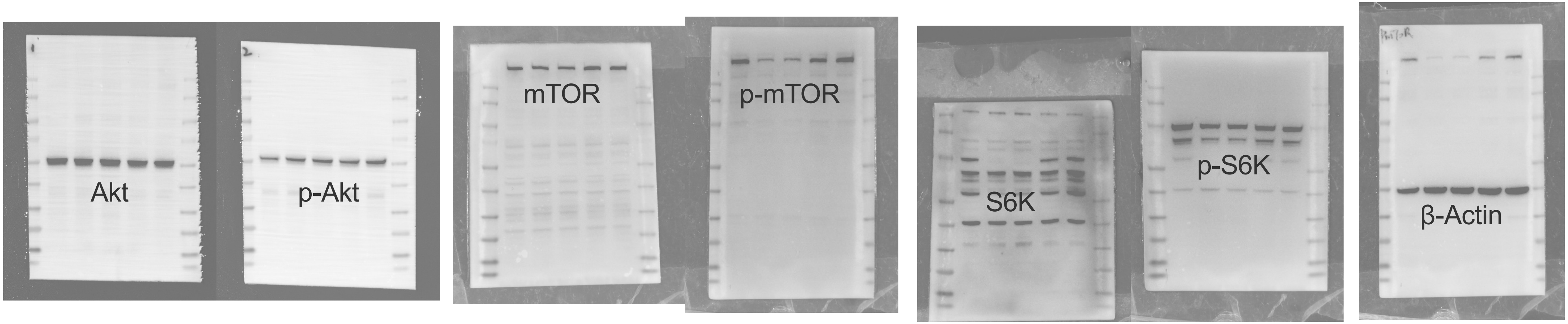

Figure 5A

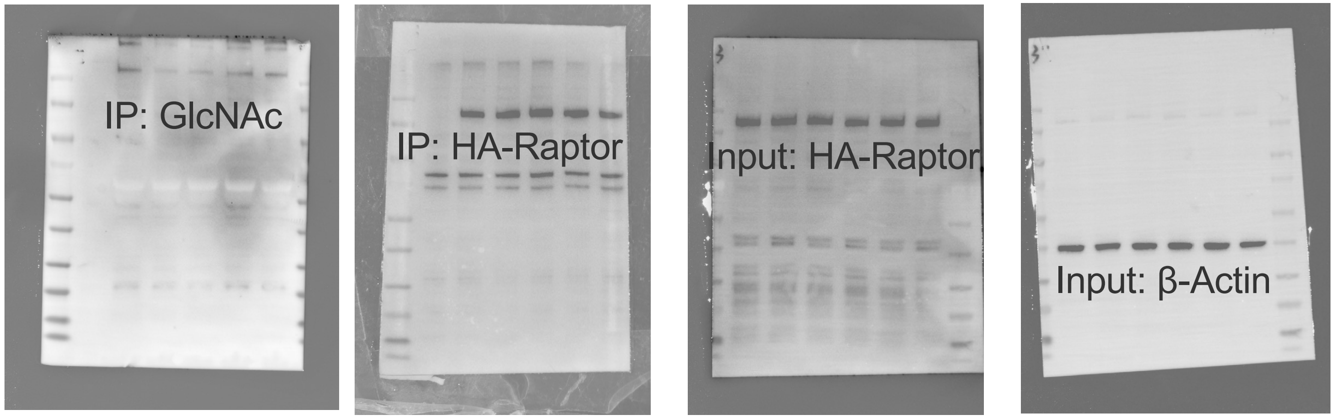

Figure 5B

Figure 5

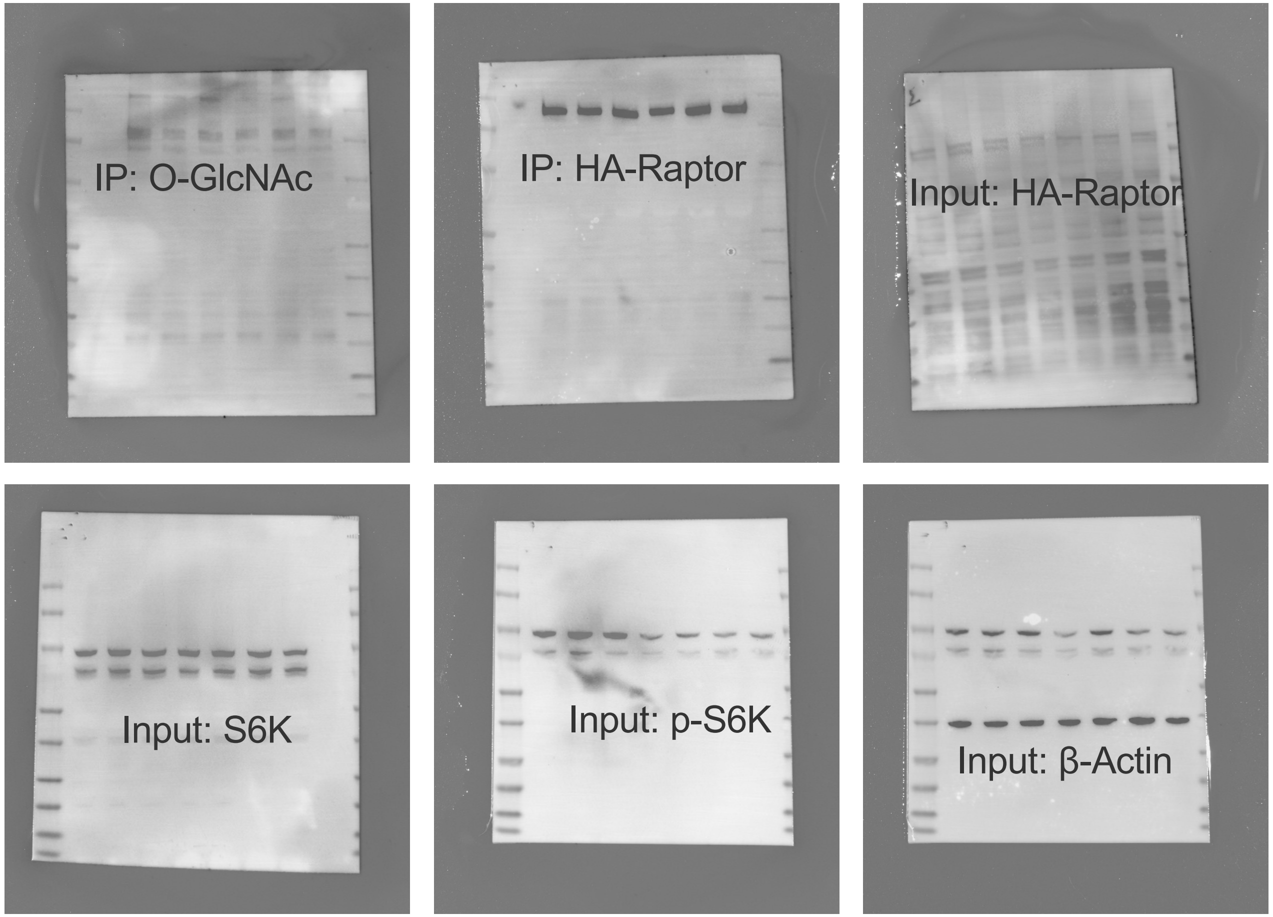

Figure 5E

Figure 5

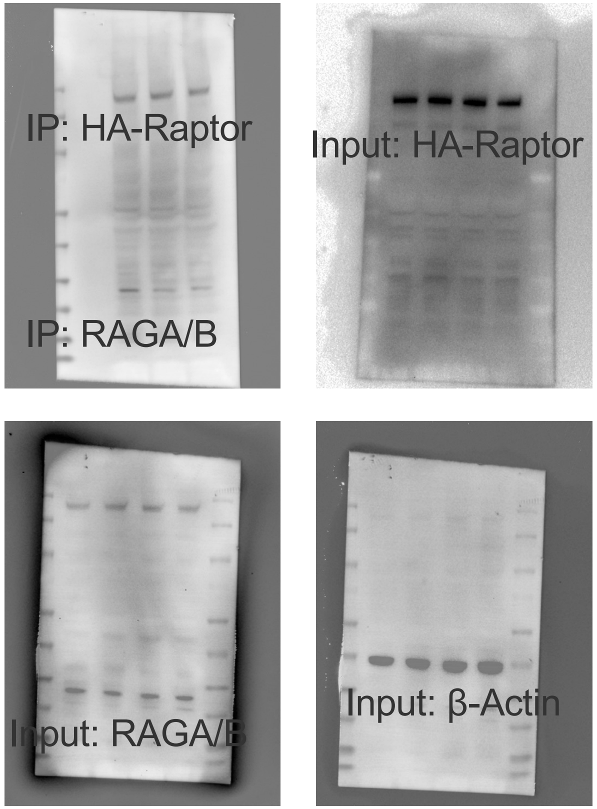

Figure 5G

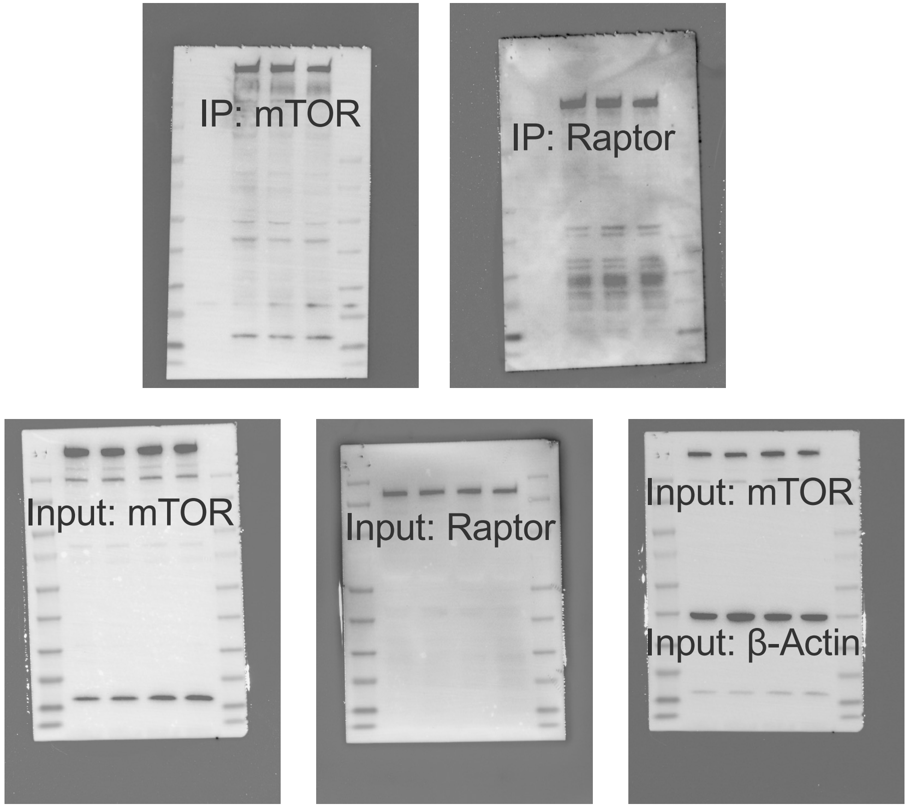

Figure 5H

Figure 6

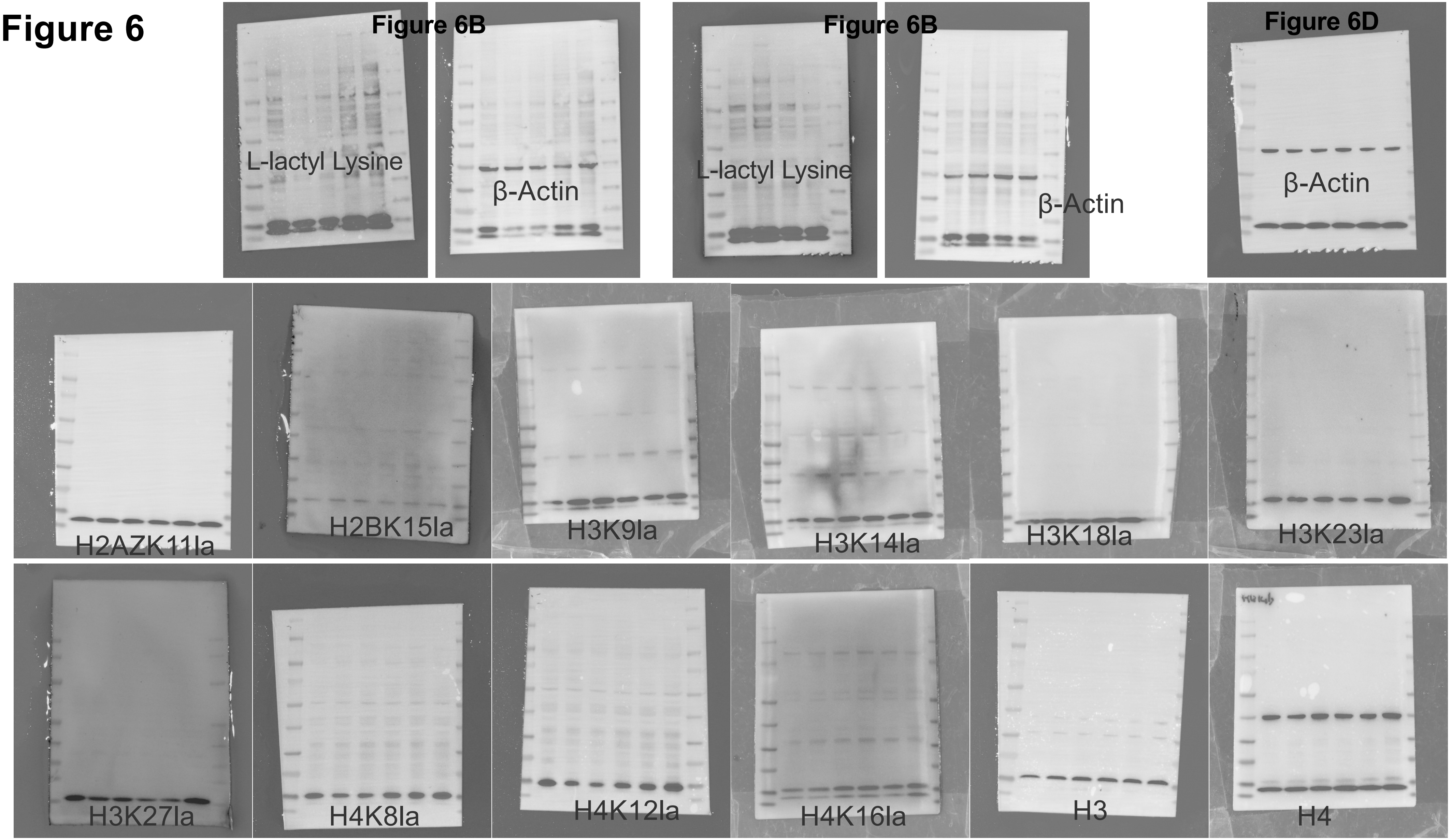
Figure 6B
L-lactyl Lysine
β-Actin
Figure 6B
L-lactyl Lysine
β-Actin
Figure 6D
β-Actin
H2AZK11la
H2BK15la
H3K9la
H3K14la
H3K18la
H3K23la
H3K27la
H4K8la
H4K12la
H4K16la
H3
H4

Figure 6

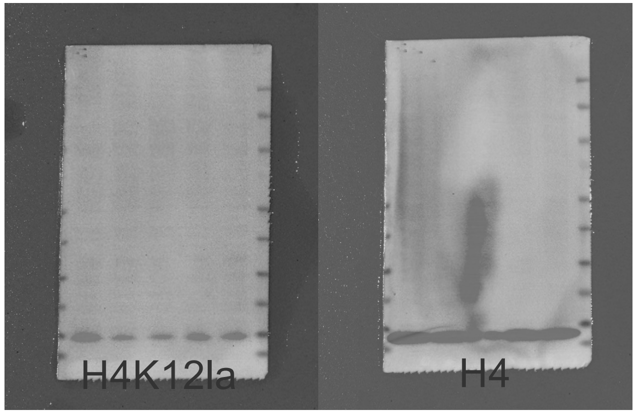

Figure 6E

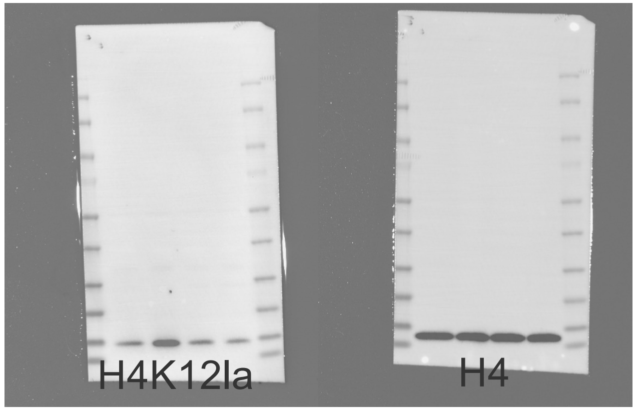

Figure 6F

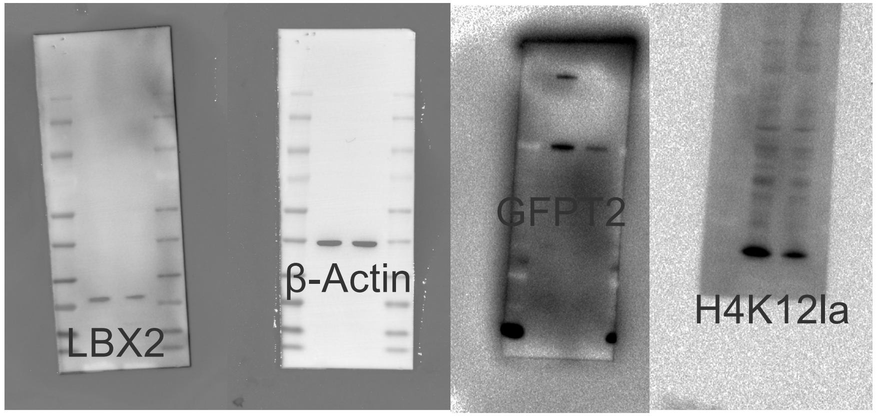

Figure 6I

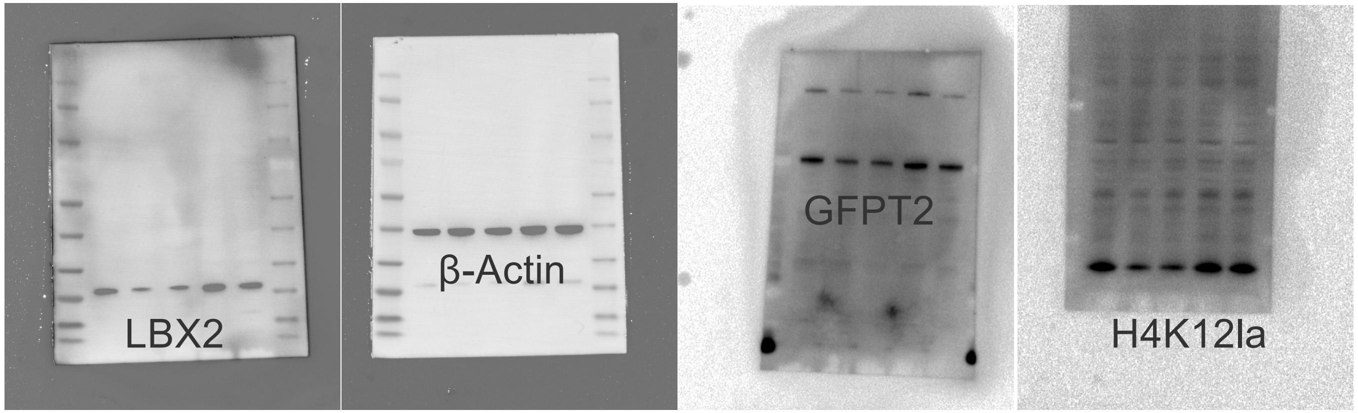

Figure 6K

Supplementary Figure 2

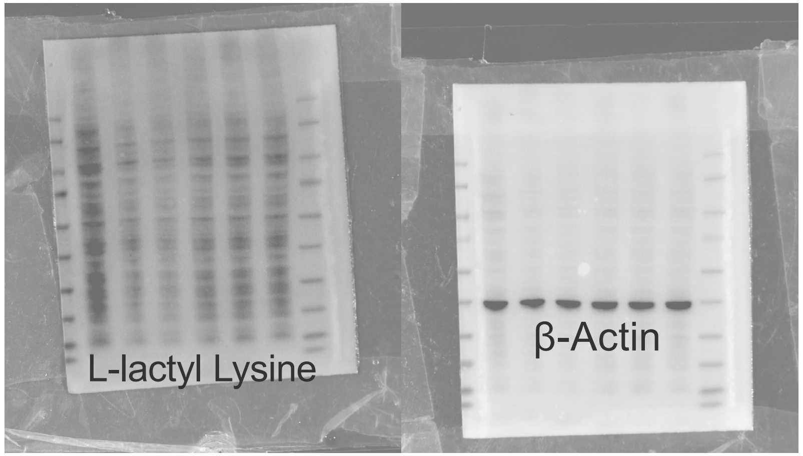

Supplementary Figure 2C

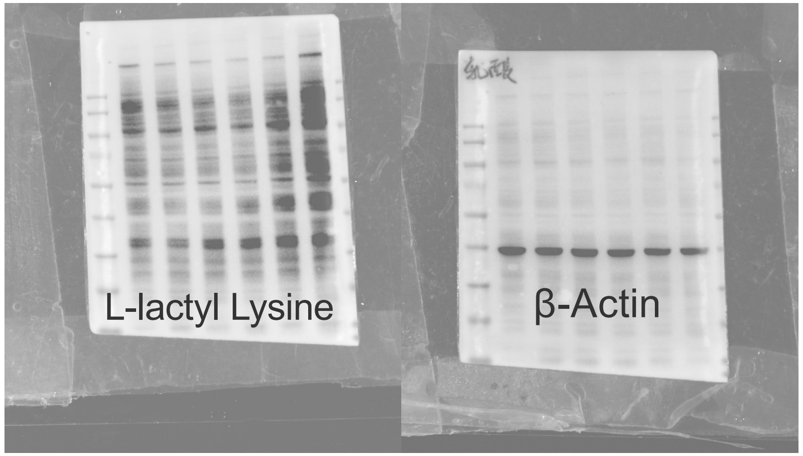

Supplementary Figure 2D

Supplementary Figure 2

Supplementary Figure 2E

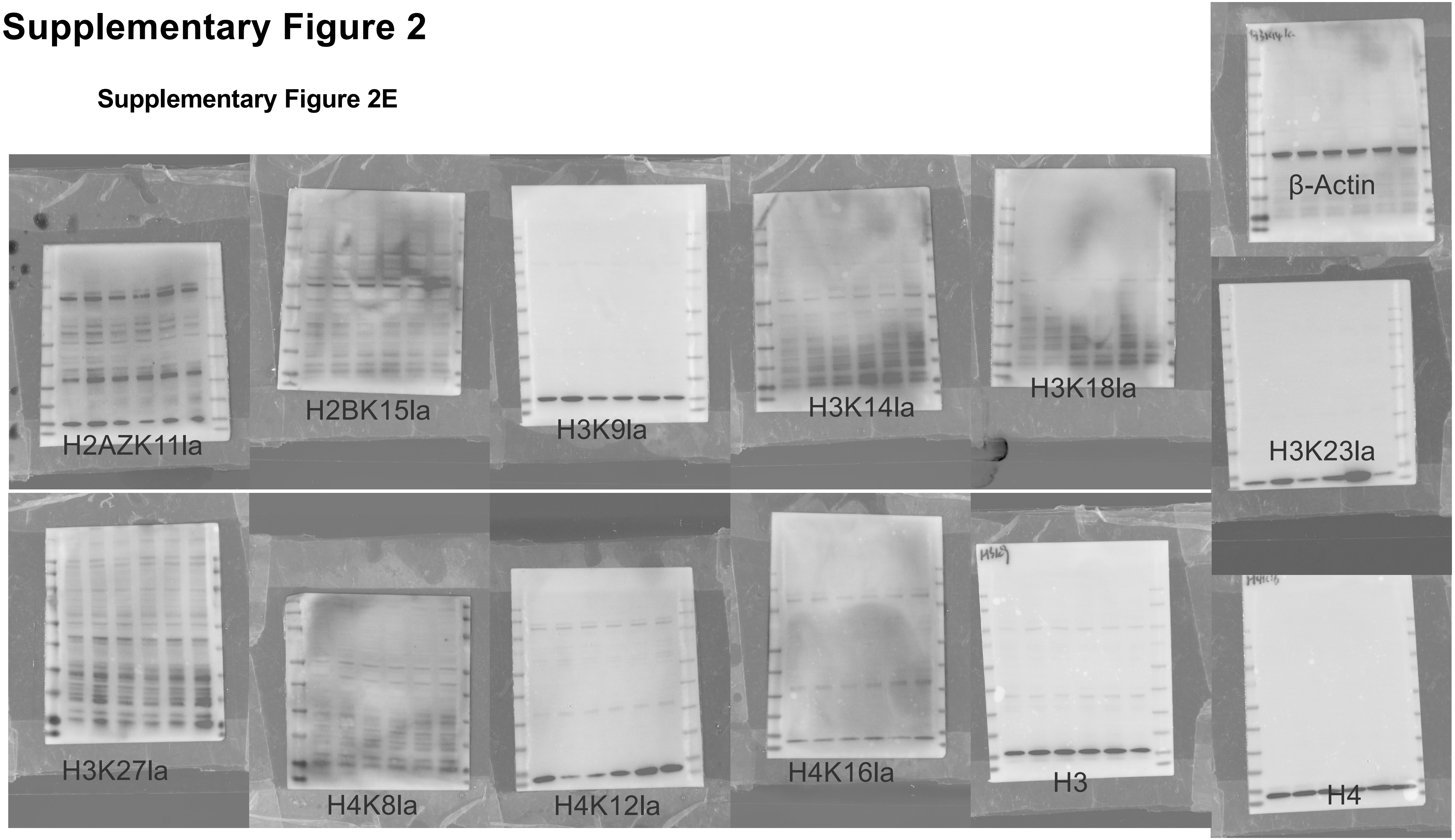

Supplement: Supplementary file 5 — Orginal Western Blot Figures [file 41420_2025_2888_MOESM5_ESM.pdf]
